# First Generation Amperometric Biosensing of Galactose with Xerogel-Carbon Nanotube Layer-By-Layer Assemblies

**DOI:** 10.3390/nano9010042

**Published:** 2018-12-29

**Authors:** Najwa Labban, Mulugeta B. Wayu, Ciara M. Steele, Tess S. Munoz, Julie A. Pollock, William S. Case, Michael C. Leopold

**Affiliations:** 1 Department of Chemistry, 138 UR Drive, Gottwald Center for the Sciences, University of Richmond, Richmond, VA 23173, USA; najwa.labban@richmond.edu (N.L.); mwayu@richmond.edu (M.B.W.); tess.munoz@richmond.edu (T.S.M.); jpollock@richmond.edu (J.A.P.); 2 Department of Biology, Chemistry, and Physics, Converse College, Spartanburg, SC 29302, USA; cmsteele001@converse.edu

**Keywords:** galactose, 1st generation biosensor, xerogel, carbon nanotube, amperometric sensor, galactose oxidase, galactosemia

## Abstract

A first-generation amperometric galactose biosensor has been systematically developed utilizing layer-by-layer (LbL) construction of xerogels, polymers, and carbon nanotubes toward a greater fundamental understanding of sensor design with these materials and the potential development of a more efficient galactosemia diagnostic tool for clinical application. The effect of several parameters (xerogel silane precursor, buffer pH, enzyme concentration, drying time and the inclusion of a polyurethane (PU) outer layer) on galactose sensitivity were investigated with the critical nature of xerogel selection being demonstrated. Xerogels formed from silanes with medium, aliphatic side chains were shown to exhibit significant enhancements in sensitivity with the addition of PU due to decreased enzyme leaching. Semi-permeable membranes of diaminobenzene and resorcinol copolymer and Nafion were used for selective discrimination against interferent species and the accompanying loss of sensitivity with adding layers was countered using functionalized, single-walled carbon nanotubes (CNTs). Optimized sensor performance included effective galactose sensitivity (0.037 μA/mM) across a useful diagnostic concentration range (0.5 mM to 7 mM), fast response time (~30 s), and low limits of detection (~80 μM) comparable to literature reports on galactose sensors. Additional modification with anionic polymer layers and/or nanoparticles allowed for galactose detection in blood serum samples and additional selectivity effectiveness.

## 1. Introduction 

Biosensor development continues to be a relevant field of research across many disciplines of science, most notably for the detection of analytes implicated in various disease states [1,2,3,4]. Biosensors differentiate a target molecule from its surrounding environment and generate a quantifiable signal proportional to the concentration of the analyte. Important components of all biosensors include analyte recognition, signal transduction, and a signal detection mechanism [5]. Biosensors utilizing electrochemical detection methods are common due to their high sensitivity, reproducibility, and ease of maintenance [1]. Popular within this realm are amperometric biosensors that utilize oxidation-reduction (redox) reactions and enzyme catalysis to generate an electrochemically measurable current signal. First-generation amperometric sensors have found widespread applications [6,7,8,9] due to their straight forward design, low cost, and ability to work with a variety of enzymes. Additionally, first-generation sensors provide more accurate readings at low concentrations when compared to other sensing mechanisms that require mediators to act as electron shuttles [1,5]. In first-generation sensors, a specific oxidase enzyme catalyzes a reaction between the target analyte and oxygen, resulting in the production of hydrogen peroxide (H_2_O_2_). The resulting peroxide is subsequently oxidized at a working electrode, and the oxidation generates a current that is directly proportional to the amount of analyte present. 

A critical feature of first-generation biosensors is the development of immobilization scaffolds that will preserve an enzyme’s structure and function. Optimal sensors must adequately immobilize an enzyme while allowing smaller analyte and product molecules to diffuse freely. While a wide array of enzymes immobilization strategies via layer-by-layer (LbL) constructs have been investigated [10,11,12], the use of xerogels in this capacity is one prominent area of research being explored as a functional component of biosensors [2,13,14]. Xerogels, formed via hydrolysis and condensation reactions between silane precursors, form a porous scaffold that is attractive for enzyme immobilization due to its chemical inertness, mechanical rigidity, and negligible swelling in solution [15]. Furthermore, xerogel porosity and permeability can be manipulated through the use of silane precursor molecules with differing R-groups (side chains). 

An additional challenge in biosensor design is ensuring high sensitivity while simultaneously maintaining selectivity for the analyte of interest. Endogenous interferent species with low oxidation potentials pose challenges to selectivity. Thus, minimization of interferent response is particularly important for clinical testing which uses whole blood or urine samples that may contain electroactive species. A common strategy employed in biosensor design is the inclusion of a semi-permeable outer layer capable of minimizing such interferent response [2,16]. A semi-permeable outer layer that has been particularly effective in LbL constructed biosensing systems has been polyurethane (PU) blends which have been shown to simultaneously prevent interference signal and improve signal-to-noise ratios [16]. The development and optimization of biosensors capable of the selective detection of small molecule diagnostic markers has important clinical applications. Previous work has focused on the detection and quantification of the biomarkers glucose, urea, and cholesterol, markers indicative of diabetes, renal failure, and heart disease, respectively [1,2,3,4].

As ad-layers of material are employed to gain selectivity of the target analyte over interferent species, sensitivity is sometimes compromised. A major strategy to combat this problem in biosensing schemes is the incorporation of nanomaterials (NMs) such as nanoparticles (NPs) or carbon nanotubes (CNTs). NMs of this nature offer unique properties with regard to biosensing including their high conductivity, favorable interaction with biological materials, and surface area–to–volume ratio. NMs have demonstrated enhancement of amperometric–based signals at electrode interfaces [15,17] including LbL constructed systems involving xerogels and semi-permeable membranes [9,18,19,20]. The collective body of work suggests that NMs provide a remedy to the trade–off between sensitivity and selectivity due their stability within the layered assemblies of these biosensors. 

In this work, the development and optimization of a first-generation amperometric biosensor scheme for the detection of galactose is presented. This monosaccharide is a primary diagnostic marker for galactosemia, a potentially lethal autosomal recessive disorder. In individuals with galactosemia, total blood galactose are >10 mg/dL (~0.56 mM), typically ranging between 5–20 mM for classic galactosemia, while normal levels are <1 mg/dL (~0.056 mM) [21]. While classic galactosemia can be are detected in the United States during newborn screening testing, forms of the disease resulting from variant base pair substitutions are not as easy to detect due to a lack of mandatory screening [21]. Furthermore, since each state determines its own acceptable cutoff values when testing, variant forms of the disease are less likely to be detected if a particular state’s cutoff value is high [22]. An easier, more accurate, and quantifiable measure of galactose could improve screening as well as monitoring. While some prior work on galactose biosensing is present in the literature [23,24,25,26,27,28,29,30,31], the number of reports is limited when compared to highly targeted species such as glucose. Prior work has also focused on the detection of galactose through gas/liquid chromatography, or electrochemical strategies requiring redox mediators (i.e., 2nd generation schemes) or the covalent attachment of galactose oxidase (GaOx) to a surface [26,29,32]. The work presented herein represents a first-generation approach to galactose detection and also builds upon previous studies that have employed first-generation sensing for the detection of other clinically relevant molecules, thus leading to the possibility of a tunable sensor template with varied detection possibilities. Moreover, the study includes an in-depth examination of the unique signal enhancement that occurs in this particular system when a semi-permeable selective membrane of polyurethane (PU) is employed and exposes the critical nature of the silane precursor used to immobilize the enzyme at the electrode. Finally, the study introduces CNTs into the xerogel/semi-permeable membranes to effectively balance the selectivity and sensitivity of an effective galactose biosensor. 

## 2. Experimental Section

### 2.1. Materials and Instrumentation

All chemicals were purchased from Sigma-Aldrich (St. Louis, MO, USA) unless specifically stated. Tecoflex SG-80A polyurethane (TPU) was obtained from Lubrizol (Cleveland, OH, USA) and Hydroethane AL25-80A polyurethane (HPU) was obtained from AdvanSource Biomaterials (Wilmington, MA, USA). Carboxylic acid functionalized single-walled carbon nanotubes (COOH-SWCNTs; 1.5 nm diameter; 1–5 μm lenght) were purchased from Nano Lab Incorporated (Waltham, MA, USA). Sheep blood serum was purchased from Hemostat (Dixon, CA, USA). All solutions were prepared using 18 MΩ·cm ultrapurified water. Amperometric current-time (*I-t*) curves were recorded with an eight-channel potentiostat (CH Instruments, Model 1030, Austin, TX, USA) and used to evaluate biosensor performance. Electrochemical cells were composed of a Ag/AgCl reference electrode (saturated KCl), Pt wire counter electrode (CH Instruments) and a modified Pt working electrode (2 mm diameter, CH Instruments). Silane precursors used in xerogel formation were stored in a desiccator and subsequently used/deposited in a relative humidity (RH)-controlled chamber (Cole-Parmer, Vernon Hills, IL, USA). UV-Vis measurements were recorded with a Jasco V-570 spectrophotometer by depositing xerogel-enzyme layers (15 μL) onto glass slides and measuring absorbance over time upon exposure to phosphate buffered solution (PBS).

### 2.2. Preparation of Sol-Gel Biosensors

Platinum working electrodes were polished successively with 1.0, 0.3, and 0.05 Al_2_O_3_ powder (Electron Microscopy Sciences, Hatfield, PA, USA). Polished electrodes were then electrochemically cleaned via cycling in 0.1 M sulfuric acid between 1.2 and −0.25 V at 0.25 V/s for 500 s. For biosensor fabrication, a solution of tetrahydrof-tran (THF, 100 μL) and silane (25 μL) was prepared in one Eppendorf tube, while galactose oxidase (GaOx, 9.4 mg) from *Dactylium dendroides* dissolved in water (75 μL) was prepared in a second tube. Each tube was shaken on a vortex for 10 min inside a RH controlled chamber (50% RH). A portion of the GaOx solution (50 μL) was then added to the silane/THF mixture, and the resulting combination was shaken on a vortex for an additional 10 min. A 3 μL aliquot of the final sol-gel mixture was deposited onto the platinum electrode surface and allowed to dry at 50% for 24–48 h. to form xerogel layers (Note: Maintaining constant RH during drying has been shown to be a critical factor in prior biosensor work) [18]. The specific silanes used in this study were octyl-trimethoxysilane (OTMS), propyl-trimethoxysilane (PTMS), methyl-trimethoxysilane (MTMS), octadecyl-trimethoxysilane (ODTMS), isobutyltrimethoxysilane (IBTMS), phenyl-trimethoxysilane (PhTMS), 3-mecaptopropyl-trimethoxysilane (MPTMS), and aminopropyl-trimethoxysilane (APTMS). 

Additional modifications to the scheme were dictated by the type of experiment being conducted. For the fundamental development of the sensors, the xerogel layers were capped with an outer polyurethane (PU)-blended semipermeable membrane which was prepared by dissolving a 50:50 mixture of hydrophilic HPU (50 mg) and hydrophobic TPU (50 mg) in ethanol and THF (2.5 mL each) which was stirred overnight. (Note: Prior work has established that the 50:50 blended PU layer is effective at discriminating against common interferents [18]) An aliquot of the resulting PU blend (10 μL) was then deposited onto the dried xerogel-coated electrode, and the assembly was allowed to dry for 30 min in the humidity chamber (50% RH) prior to evaluation as a sensor. 

Selective membranes were also incorporated into the more effective schemes. For the electropolymer semi-permeable diaminobenzene and resorcinol (DBR) membrane underlayer, a procedure was adapted from prior work [24]. Briefly, monomers of each component (1.5 mM) were dissolved in N_2_ saturated 4.4 mM PBS at pH 7. The solution was used immediately after preparation, protected from light, and blanketed with N_2_ during the electropolymerization process. Clean platinum electrodes were placed in the monomer mixture and the potential was cycled 10 times from +0.03 V to +0.83 V at 2 mV/s. The DBR-modified electrodes were further augmented with deposition of 5 μL of COOH-SWCNTs mixed with 1 wt. % Nafion solution at 1 mg/mL solution, previously mixed under sonication (1 h). As described below in the Results and Discussion Section, the Nafion solution was also infused into outer layers to give the assembly additional anionic character to add selectivity.

### 2.3. Evaluation of Galactose Biosensor Performance

Before testing, prepared biosensors were soaked in PBS buffer solution (pH = 6.5 or 7.0, 8 mM) for 1 h at room temperature to ensure xerogel hydration. After soaking, the sensors were placed in fresh PBS buffer (25 mL) and stirred at a constant rate of 1100 rev/min for current-time (*I-t*) experiments. The working electrode potential was held at +0.65 V for 1200 s before successive injections (25 μL) of 1 M galactose solution at 100 s intervals. The results were saved as amperometric *I-t* curves and converted into calibration curves of current response as a function of galactose concentration. The current associated with a certain galactose concentration was calculated by averaging the current from 80–100 s after the injection to ensure adequate time for the sensor to stabilize. 

Sensor sensitivity to galactose was based off of linear regression analysis of the calibration curves for sensor current response at increasing galactose concentrations up to 10 mM. Response time (*t*_r-95%_) was conservatively defined as the time elapsed until 95% of the total response was recorded. 

## 3. Results and Discussion 

### 3.1. Galactose Sensitivity 

**Scheme 1:**
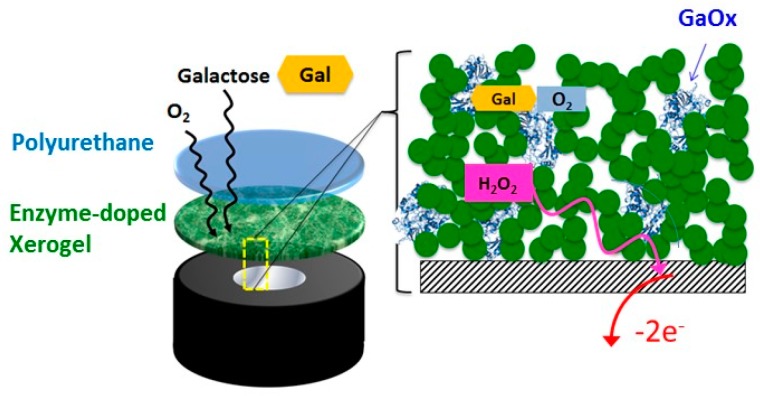
1st Generation Biosensing Scheme.

The general strategy of our biosensor design is illustrated in Scheme 1 and features layer-by-layer (LbL) construction at a platinum electrode with a sol-gel layer embedded with enzyme and capped with a semi-permeable membrane of blended polyurethane (see Experimental Section). 

**Figure 1:**
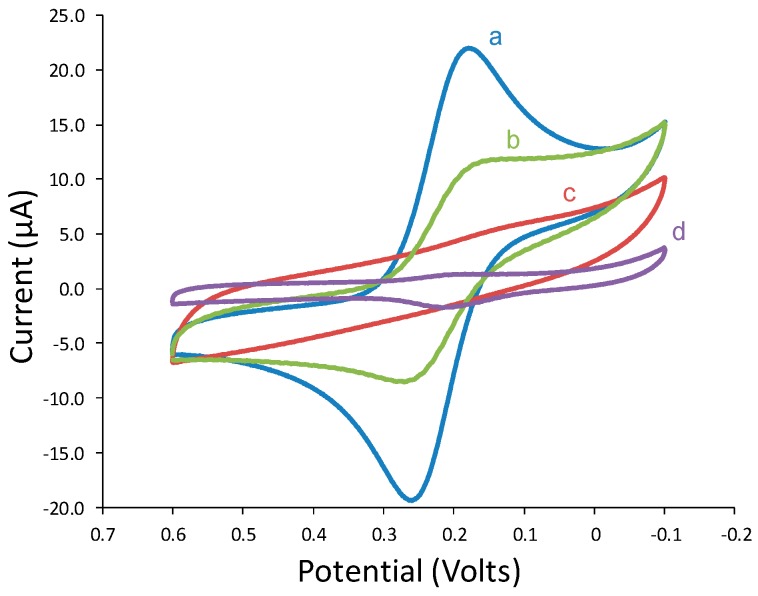
Cyclic voltammetry of 5 mM potassium ferricyanide (1 M KCl) at (a) bare platinum electrodes, platinum electrodes modified with (b) OTMS xerogel embedded with GaOx, (c) OTMS xerogel embedded with GaOx and an added layer of polyurethane (50:50 HPU:TPU), and (d) single layer of polyurethane. Scan rate: 100 mV/s.

**Figure 2:**
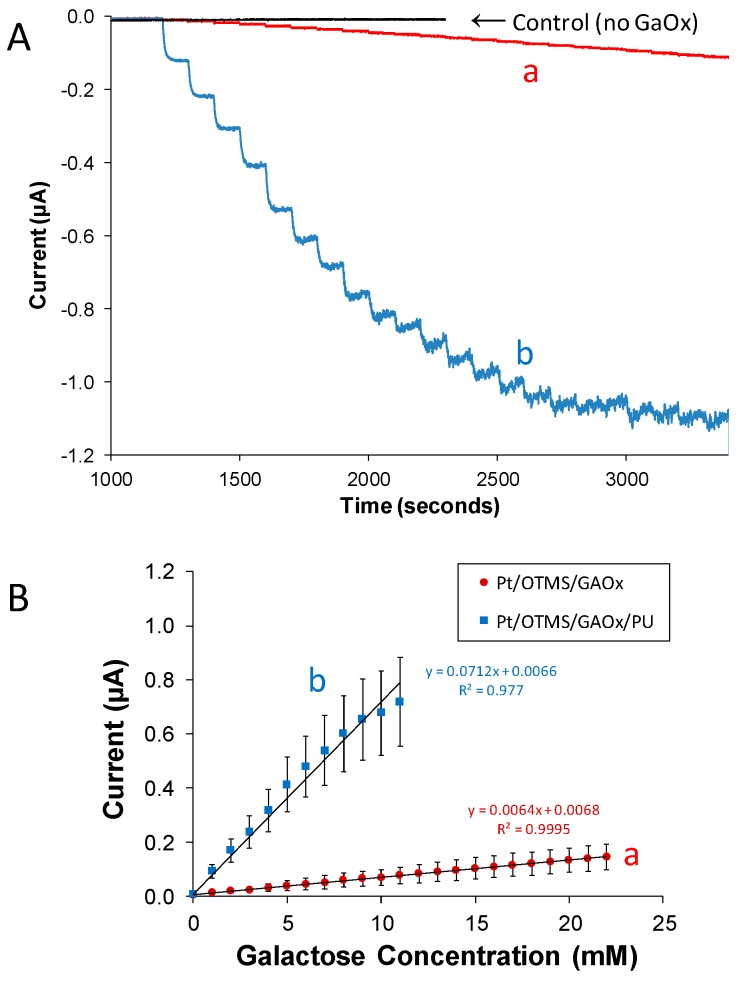
Representative (**A**) amperometric *I-t* curves and (**B**) corresponding calibration curve analysis during 1 mM injections of galactose at (a) Pt/OTMS (GaOx) and (b) Pt/OTMS GaOx)/PU. The *I-t* responses of these systems are also compared to a control system without enzyme and PU capping layer (Pt/OTMS—no GaOx). Solution conditions: 8 mM PBS (pH 7); error bars represent standard deviation.

This general scheme has been successfully employed in previous studies on a glucose model system [2] as well as for uric acid sensing [13]. The scheme was first constructed, as described in the Experimental Section, using an OTMS xerogel layer capped with the blended PU at a platinum electrode. As in other work studying similar materials [2,9,13], the successful deposition of these layers was independently verified with cyclic voltammetry probing experiments of ferricyanide at each interface to observe the subsequent blocking of solution redox species at modified electrodes. Both the successful initial deposition of the xerogel layer and subsequent capping of the PU layer at the platinum electrode are confirmed with the depression of the ferricyande probe molecule cyclic voltammetry at these modified electrodes (Figure 1). Upon immersion into buffer and subsequent injections of galactose while under potential control (+0.65 V), amperometric responses in the form of a stair-case current-time (*I-t*) curve were clearly observed (Figure 2A).

In these results, the injection of the galactose substrate resulted in the apparent production of peroxide via an enzymatic reaction. The peroxide was then subsequently and immediately oxidized at the electrode interface to yield a sharp increase in anodic current and the expected stair-step response. As a control, injections at xerogel-modified electrodes without immobilized GaOx exhibited no current response. *I-t* curves can be translated into calibration curves (Figure 2B) which show a linear response to galactose concentrations that easily span the relevant clinical diagnosis range (0.056 to 0.56 mM). The presence of peroxide was verified by repeating the experiment but adding catalase, a peroxide-consuming enzyme, to the test solution after multiple injections of galactose. Upon injection of the catalase, the step response immediately reverses with lower current observed indicating a decreased concentration of peroxide in the solution (Supplementary Materials, Figure SM-1). Similar results were achieved using platinum electrodes modified with xerogel layers of either PTMS or IBTMS and capped with PU (Supplementary Materials, Figures SM-2–4).

With other schemes of this nature, the addition of any material to an electrode, but particularly the outer PU membrane, typically results in more defined stair-step current responses at the expense of lower sensitivity (i.e., smaller step currents and lower slope of the corresponding calibration curve) [2,13]. This type of signal attenuation is not unexpected when additional materials are used to modify—a result typically observed when one modifies an electrode with additional material. Interestingly, in the galactose oxidase system, the addition of a PU outer layer generally resulted in less noise (a more defined amperometric response and a substantially higher sensitivity (slope) when compared to electrodes without PU (Figure 2B). This result was highly repeatable and was also observed at the systems using PTMS and IBTMS as well (Supplementary Materials, Figures SM-3 and SM-4). This result, the enhancement of signal with the addition of the PU ad-layer, stands in stark contrast to prior work on xerogel-based biosensor schemes of this nature and is explored further in a later section to understand the factors contributing to this effect. 

The amperometric response of the PU-capped sensors using GaOx-doped OTMS, IBTMS, and PTMS was very robust. Stability studies examined the system’s ability to maintain sensitivity levels after multiple uses of modified electrodes. PU-capped enzyme-doped OTMS, PTMS, and IBTMS xerogel modified electrodes were stored for 24 h in PBS (pH = 7.0) at 5–7 °C and subsequently measured for galactose sensitivity. After each measurement, the modified electrodes were stored under the aforementioned conditions for an additional 24 h. Biosensor performance after multiple uses was determined by calculating the sensor’s percent function after each run according to Equation (1), where *S_n_* is the sensitivity of a given run, *n*, and *S_i_* is the sensitivity of the initial run:

(1) $$F\left(t\right)=100+\left(100\left(\frac{S_{n}-S_{i}}{S_{i}}\right)\right)$$

Overall, the results of these stability experiments, shown in Supplementary Materials (Figure SM-5), suggested an initial decay of sensitivity before stabilizing between 20% and 60% of the original function depending on the type of xerogel. This retention is acceptable considering the relevant physiological concentration of galactose to be detected is so low (0.056 to 0.55 mM). While the sensors remained effective after nearly 10 days, the most notable decrease in sensitivity seemed to occur after 5 days of usage. It is possible that the initial decay and eventual plateau is representing experimental evidence of the continued cross-linking that occurs with the silane precursors and consequential changes to the porosity of the films. 

### 3.2. Polyurethane Enhancement Effect

Within the general scheme, the effects of individual variables (silane precursor, buffer pH, drying time, and enzyme concentration) were systematically examined to understand the unique enhancement effect observed with the addition of PU to these systems. First, the effect of buffer pH was studied given its influence on enzyme function and its importance in using the sensors in biological fluid (blood, for example, exhibits a typical pH of 7.4). Considering pH effects, prior work has suggested that GaOx exhibits optimum catalytic efficiency as pH increases from 6.5 to 7.5, with decreases in activity observed above pH 7.5 [17]. This research found that for electrodes modified with both OTMS and PTMS, xerogels that both exhibited increased sensitivity with the addition of PU, adjusting the pH from 6.5 to 7.0 did not result in significant changes in sensitivity (Supplementary Materials, Figure SM-6). The results suggest that small adjustments in pH within the enzyme’s optimal range are not having a significant impact. 

**Scheme 2:**
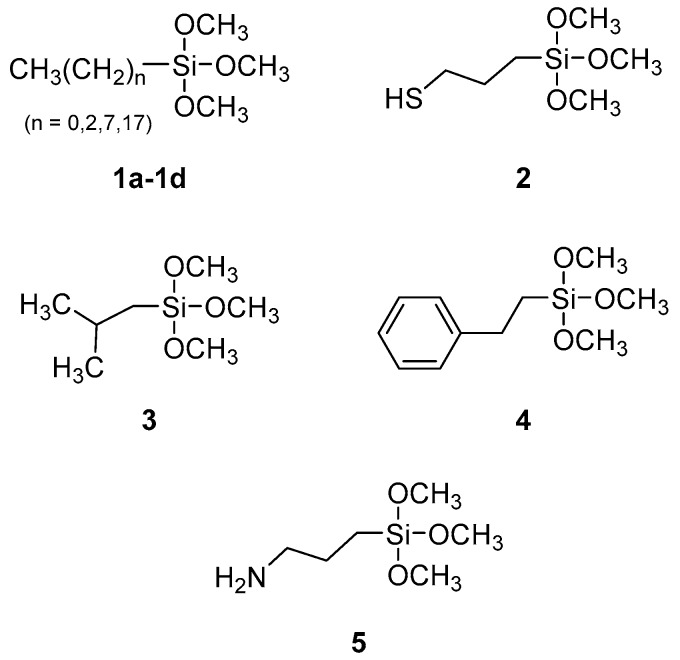
Xerogel Silane Precursors.

Enzyme concentration and drying time, the latter affecting film porosity/permeability, have been previously shown to influence the effectiveness of similar xerogel-based biosensing schemes [2,18]. For drying (i.e., aging), GaOx-doped xerogels were investigated after being dried and aged for both 24 and 48 h. The results for the OTMS and PTMS suggest that there was an inconsequential difference in galactose sensitivity for the two different aging times (Supplementary Materials, Figure SM-6). Similarly, GaOx enzyme concentration was explored to see if sensitivity would be enhanced at higher concentrations. Doubling the enzyme concentration (from 5.4 mg/mL to 10.8 mg/mL) used in modification of the PTMS and OTMS systems also did not result in significant improvement in galactose sensitivity (Supplementary Materials, Figure SM-6). 

**Figure 3:**
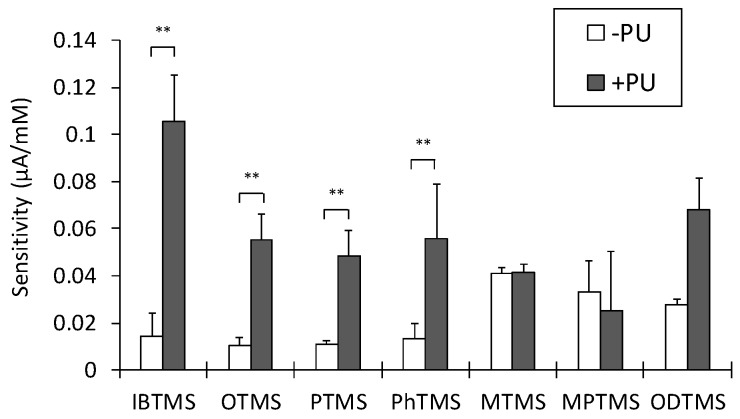
Comparison of galactose sensitivities for electrodes modified with various GaOx-doped xerogels with (+PU) and without (-PU) the PU capping layer. For each silane without and with PU, the number of electrodes were IBTMS (3, 5), OTMS (7, 8), PTMS (10, 11), PhTMS (6, 6), MTMS (6, 6), MPTMS (5, 6), and ODTMS (3, 3). Data presented as the average with standard error. For each pair, a Welch’s *t*-test was performed; ** *p* value < 0.1.

A variety of silane precursors (Scheme 2) were studied for their viability as immobilization matrices for GaOx and to see if the type of xerogel affected the PU signal enhancement. Of the silane precursors tested, only electrodes modified with APTMS (**5**) failed to detect galactose at all. This PU effect of increasing sensitivity was observed only with specific silane precursors: OTMS (**1c**), PTMS (**1b**), IBTMS (**3**), and PhTMS (**4**). It is notable that these particular silane precursors all possess hydrocarbon-based R-groups. In contrast, other silane precursors used to form GaOx-doped xerogels, including MTMS (**1a**), MPTMS (**2**), and ODTMS (**1d**), did not exhibit an enhancement effect with the addition of the PU capping layer. It should be noted that xerogels formed from representative silane precursors from both these groups were also examined for pH, GaOx concentration, and drying time dependence, including ODTMS, MTMS, and PhTMS xerogel systems. As with the other systems, the results suggest that none of these factors have significant impact on galactose sensitivity (Supplementary Materials, Figure SM-7). A summary analysis of galactose sensitivity as a function of silane precursor used to create the xerogel layer with and without PU is shown in Figure 3. For each system without and with PU, a Welch’s *t*-test was performed; significant differences were observed for IBTMS, OTMS, PTMS, PhTMS (*p* = 0.0076, 0.0045, 0.0055, 0.0043, respectively) while no significant differences were observed for MTMS, MPTMS, and ODTMS (*p* = 0.9182, 0.5778, and 0.2138, respectively). The number of ODTMS films tested were limited in this study with the results displaying high variability that resulted in a *p* value that, while lower than MTMS and MPTMS, still showed no statistically significant difference. The ODTMS results are not entirely unexpected based on prior work where such films were found to have low porosity/high hydrophobicity and, ultimately, lower sensor sensitivity—all results likely from the significantly longer octadecyl hydrocarbon chain. Overall, the data support the conclusion that xerogels formed from silanes containing medium, aliphatic side chains result in sensors with higher sensitivity upon inclusion of a PU outer layer. A summary of galactose sensitivity parameters for the PU-capped systems OTMS, PTMS, IBTMS, and Ph-TMS is provided as Table 1. 

**Figure 4:**
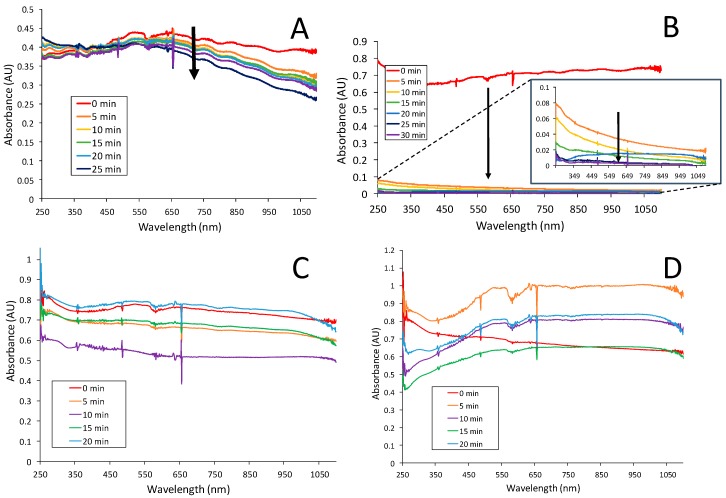
UV-Vis spectra of GaOx-doped xerogels during prolonged exposure (soaking, 20–30 min.) to 8 mM phosphate buffer (pH = 6.5) including (**A**) MTMS, (**B**) OTMS, (**C**) MTMS with PU capping layer, and (**D**) OTMS with PU capping layer. Notes: OTMS without PU shows an immediate (within 5 min) decrease in absorbance that continues over time ((**B**), Inset). Spectrum of GaOx solution (control) is included in Supplementary Materials, Figure SM-8.

While the PU enhancement effect was clearly established for certain systems, the source of the effect was not well-understood. It should be noted that adjusting the ratio of HPU to TPU for outer blended PU layer did not yield significant enhancement for any of the systems (results not shown), suggesting that the hydrophobic/hydrophilic nature of the outer layer is less significant than its physical interaction with the enzyme-doped xerogel layer and the testing environment. It was hypothesized that the significant sensitivity enhancement provided by the PU outer layer in these systems may be related to the layer’s ability to mitigate GaOx enzyme leaching, particularly given the smaller size of GaOx compared to other oxidase enzymes that have been embedded in xerogels [2,13,16]. In that prior work with other enzymes (e.g., glucose oxidase, uricase), dramatic increases in sensitivity with the addition of PU capping layers were notably not observed. To test this hypothesis, representative films of GaOx-embedded xerogels that exhibited either significant (OTMS) or insignificant (MTMS) enhancement with PU capping were spectroscopically studied by forming the films on glass slides and observing their UV-Vis spectra to see the effects of exposure to PBS over ~30 min. Briefly, xerogel films comprised of these two silanes, each doped with GaOx, were formed on glass slides under the same conditions as the modified electrodes used in the biosensors. The slides were then immersed in PBS and periodically removed, dried, and measured with a UV-Vis spectrophotometer. Additional experimental details on these specific procedures are provided in the Supplementary Materials. Because the films are formed and soaked without a PU layer, they are potentially susceptible to GaOx leaching from the film, an effect that would result in decreasing absorbance over time. As shown in Figure 4, the spectra for GaOx-doped MTMS upon exposure to PBS are relatively consistent over time suggesting that the enzyme content of the film remains relatively constant (minimal leaching). The OTMS xerogel, on the other hand, shows a significant decrease in absorbance almost immediately upon exposure to solution (Figure 4B), a trend that continues more slowly after 5 min of exposure up to 30 min of exposure (Figure 4B, inset). This result suggests that, without the PU layer, there is extensive leaching of the enzyme from the OTMS matrix. Analogous films of MTMS and OTMS that are capped with PU do not show this effect (Figure 4C,D). 

**Figure 5:**
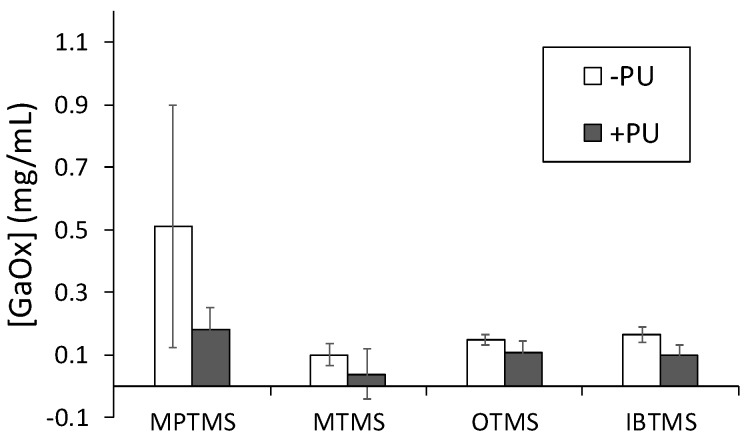
BCA assay (562 nm) results quantifying amount of leached GaOx from xerogels with (+PU) and without (-PU) the PU capping layer including MPTMS and MTMS, which exhibit no PU-based sensitivity enhancement, as well as OTMS and IBTMS, which exhibit PU-based sensitivity enhancement (*n* = 3).

Protein quantification assays (Bicinchoninic acid (BCA) assays) were conducted to provide more quantitative data in regards to the enzyme leaching. Experimental details for the assay experiment and analysis is provided in the Supplementary Materials. Briefly, xerogel films comprised of representative silane precursors that either exhibit sensitivity enhancement (OTMS and IBTMS), or do not enhance sensitivity (MPTMS and MTMS), were deposited on electrodes and half of them were capped with PU. The films with and without the PU capping layer were then exposed to PBS for one hour before aliquots of the soak solution were transferred to an assay plate. The amount of leached GaOx in the solutions contacting the films was quantified by comparing to a standard curve as shown in Figure 5. Analysis using a 2-sample Welch’s *t*-test determined that the difference associated with the addition of PU was more significant for IBTMS and OTMS than for MPTMS and MTMS (*p* = 0.0592 < 0.1477 < 0.2198 < 0.2862, respectively). The statistical analysis is not as robust with this method due to the experimental set up and smaller sample size; however, the trend of enzyme leaching is consistent with the electrochemical data. That is, the addition of PU to the IBTMS and OTMS xerogels seems to aid with the retention of GaOx, which is leached to a greater extent from these xerogels than MPTMS and MTMS gels, effectively resulting in the observed elevation in the sensitivity of the sensor due to increased metabolic activity at the electrode surface. Taken collectively, the BCA assay and UV-Vis spectroscopy results suggest that the xerogels of OTMS and IBTMS, where the sensitivity enhancement is observed, are more porous than the xerogels where the PU effect is not observed (MPTMS and MTMS)—a result that is comparable to prior characterization of these films. The greater porosity of the films allows for more substantial leaching, particularly for a smaller enzyme like GaOx, and necessitates the PU capping layer. The larger porosity of the more sensitive films may also contribute to a film structure environment more conducive to substrate/peroxide diffusion (Scheme 1) [8]. These results collectively point out the critical nature of selecting an appropriate xerogel matrix in order to gain sensitivity toward an analyte. 

### 3.3. Galactose Biosensing Performance Using Nanomaterials and Polymer Layers

Sensitivity toward galactose was achieved with the GaOx xerogel layer and enhanced with the addition of the PU capping layer but effective biosensor design promotes both sensitivity and selectivity toward the targeted analyte. Even though selectivity is critical for biosensing performance, it is not uncommon for reported biosensor designs in the literature to either insufficiently address interferent response [26,27,31]. For the xerogel-based, LbL-constructed 1st generation biosensor design in this study, some selectivity is achieved with the enzymatic reaction, the choice of xerogel (i.e., silane precursor), and the PU blend comprising the capping layer. However, more effective selectivity is often achieved in such systems with the addition of a semi-permeable membrane, often an electropolymer, to achieve discrimination against specific interferent species [2,13,16]. The PU-capped OTMS and PTMS xerogel systems in this study, while sensitive toward galactose, were also found to be susceptible to specific interferents as observed in the amperometric *I-t* curves of these systems (Supplementary Materials, Figure SM-9), with and without the PU capping layer, during injections of both galactose as well as common endogenous interferent species (e.g., ascorbic acid (AA), sodium nitrite, UA, oxalic acid, and glucose). In these results, the well-established, traditional effect of the PU capping layer is easily observed—a significant improvement in the noise associated with the recorded amperometric signal [2,13]. However, the results also illustrate that the PU-capping layer and different xerogels are insufficient for selectivity against both AA and UA. These species are often problematic interferents as both are electroactive and undergo oxidation at the applied potential (+0.65 vs. Ag/AgCl, satd. KCl). Both species are anions at the pH of the working solution (~7) suggesting that any additional layers added to the system for selectivity should increase the anionic character of the film to discriminate against urate and ascorbate. 

While many polymeric layers are available for this type of application [33], a prior study with galactose suggested that the incorporation of anionic Nafion and a copolymer of diaminobenzene and resorcinol was effective for both AA and UA discrimination while allowing for substantial H_2_O_2_ diffusion [24]. As described in the Experimental Details Section, procedures were developed to apply the copolymer of diaminobenzene and resorcinol (DBR). However, as seen in prior work [2,13], the application of additional layers, such as a semi-permeable polymer layer, often leads to significant signal attenuation and sacrifices sensitivity. The LbL construction of these xerogel-based films, however, allows for the use of nanomaterials such as nanoparticles [9,18] or carbon nanotubes (CNTs) [19,34] that can significantly enhance the targeted analyte signal in the presence of the selective polymeric membranes. 

In designing an effective galactose biosensor based off of the materials and strategy in this study, GaOx-doped xerogels of IBTMS were selected as they showed the highest sensitivity in the PU capped systems (Table 1). Platinum electrodes were first modified with a DBR electropolymer layer (See Experimental Details) before COOH-SWCNTs were dispersed with and without 1% Nafion in ethanol solutions. Both films were subsequently modified with IBTMS xerogels doped with GaOx and an outer PU layer.

**Figure 6:**
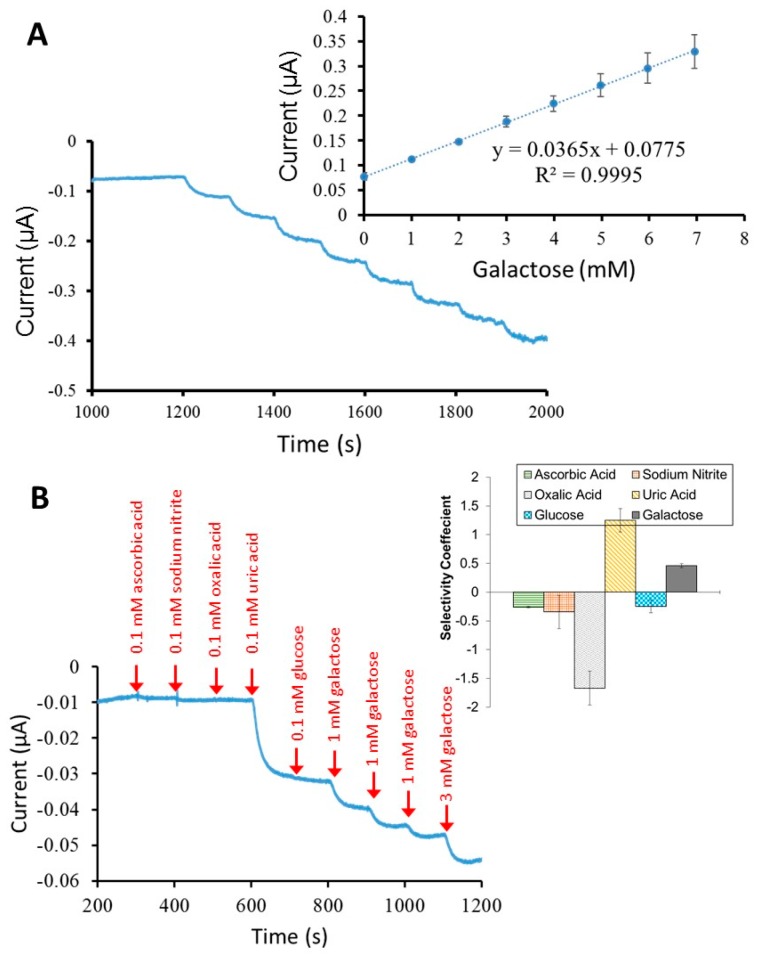
(**A**) Representative amperometric *I-t* curve and corresponding calibration curve (inset) during 1 mM injections of galactose and; (**B**) representative amperometric *I-t* curves and corresponding selectivity coefficient graphs (inset) of Pt/DBR/COOH-SWCNT (1% Nafion)/IBTMS(GaOx)/PU during injections of common interferent species and galactose in 100 s intervals. Note: In some cases, error bars are smaller than markers (*n* = 4).

Figure 6A shows the *I-t* and calibration curve collected during galactose injections for the system constructed using the Nafion membrane to disperse the CNTs. Comparing this result to the same experiments conducted without employing the Nafion layer (i.e., COOH-SWCNTs dispersed without Nafion—Supplementary Materials, Figure SM-10), it reveals the expected effects of incorporating both the Nafion and the COOH-SWCNTs; the incorporation of COOH-SWCNTs produces the highest sensitivity of any system tested (0.245 μA/mM) but the addition of the Nafion layer dampens that enhancement (0.036 μA/mM), though still maintaining effective sensitivity. The limit of detection (LOD) is 78 μM and the lower limit of quantification is 260 μM, both of which are well below the diagnostic levels for galactosemia (≥0.5 mM) [21].

While the decrease in sensitivity with the addition of Nafion is an undesired effect, it is justified if greater selectivity can be achieved. In Figure 6B, *I-t* curves during injections of common interferent species at systems using Nafion used to disperse the COOH-SWCNTs show the substantial impact the polymer layer has on certain interferent response, particularly discrimination against ascorbic acid. As in previous studies [9,16,35,36], selectivity coefficients (*K_j_^amp^*) were calculated to provide conservative quantitative assessment of selectivity as has been done in previous studies using the following equation:

(2) $$K_{j}^{amp}=\log\left(\frac{\Delta I_{j}/C_{j}}{\Delta I_{galactose}/C_{galactose}}\right)$$

where ∆*I_j_* and ∆*I_g_* are the measured currents for a specific interferent species (*j*) and galactose and *C_j_* and *C_g_* are concentrations of the interferent species and galactose, respectively. Negative values of *K_amp_* indicate that the interferent is inconsequential (i.e., no significant response compared to analyte response) whereas species with near zero or slightly positive values are selected for by the sensor. The selectivity coefficient graph (Figure 6B, inset) reiterates the effective discrimination of the common interferents with the addition of the Nafion when compared to analogous systems not employing the Nafion layer (Supplementary Materials, Figure SM-11). While the system, as presented, is not optimized toward uric acid discrimination, the demonstrated effectiveness of employing the anionic Nafion layer to eliminate ascorbate suggests that minor modifications to the LbL approach, including further incorporation of Nafion or other anionic polymers or nanomaterials [37], perhaps in the PU layer itself would be effective. Preliminary results of the system where citrate-stabilized gold nanoparticles (CS-NPs) were infused into an additional PU layer to add anionic character at the solution interface showed more effective discrimination of urate (Supplementary Materials, Figure SM-12), though the use of gold NPs in this manner requires additional study. 

**Figure 7:**
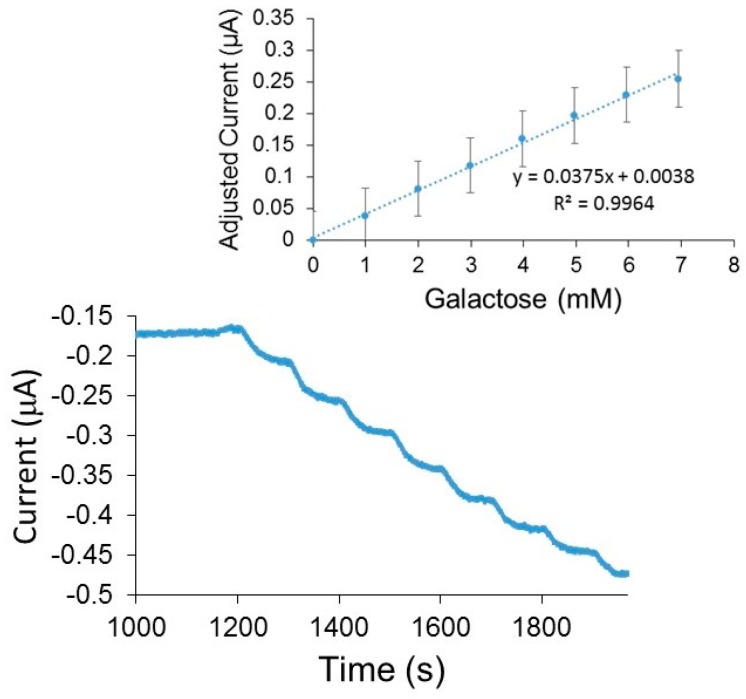
Representative amperometric *I-t* curve and corresponding calibration curve (inset) during 1 mM injections of galactose at Pt/DBR/COOH-SWCNT (1% Nafion)/IBTMS(GaOx)/PU w/Nafion in blood serum samples (50:50 *v*/*v* with PBS).

A critical aspect of any potential biomedical device of this nature is its effective operation in bodily fluids. In the case of galactosemia diagnostic tests, the media most likely to be screened is blood or blood serum. Figure 7 shows representative *I-t* curve and calibration curve for an optimized film in blood serum samples and shows that galactose sensitivity, linear range, LOD (112 μM) and the lower limit of quantification (373 μM) are maintained for effective diagnostic galactose concentrations. Calibrated sensor constructs were used on a limited number of blood serum samples spiked with 2 mM galactose (Supplementary Materials, Figure SM-13) and showed an average percent recovery of nearly 90% (relative average error −12 (±3)%; *n* = 3). 

The performance of the sensors designed in this study equal or exceed published reports on amperometric galactose biosensors [23,24,25,26,27,28,29,30,31], though parameters and measurements are often difficult to compare across studies which involve different media, testing procedures, and often do not define how a parameter was measured (e.g., few report selectivity coefficients). A table of comparison is provided in Supplementary Materials (Table SM-1). The most problematic issue with previously reported galactose biosensors of this nature appears to be that the linear range is often misaligned with the most useful diagnostic concentrations (e.g., 0.5 mM to 10 mM), with reports showing galactose detection at either too high [26,29] or too low [23,28,30] of a concentration range. A number of the reports also do not report galactose sensitivity [23,24,25,26,27,28,29,30,31,32,33,34,35,36], test in blood media [26,27,29,31] or test for discrimination of common intereferents [23,27,28,29,31]. While some sensors are comparable to the system described here, our study benefits from fundamental exploration trying to understand the functionality of the xerogel enzyme immobilization matrix and semi-permeable membranes as well as the incorporation of nanomaterials for signal enhancement—all of which, if understood, could allow for adapting the strategy and materials of this nature to other targets of interest.

## 4. Conclusions

The design of a first-generation galactose biosensor utilizing xerogel matrices, CNTs for signal enhancement, and polymeric layers for selectivity has been shown to exhibit high sensitivity, clinically relevant limits of detection and linear sensing ranges, as well as discrimination against common interferents. While the presence of the outer PU layer enhanced galactose sensitivity, the study revealed that the choice of silane precursor for the xerogel matrix was critical for retaining immobilized enzyme to achieve the enhancement. This work suggests that minimization of enzyme leaching is particularly important when incorporating smaller enzymes into 1st generation schemes of this nature. The addition of polymers to the LbL construction is effective for gaining discrimination against interferents while the use of CNTs compensated for the loss of sensitivity with the addition of selective layers. Taken together with other biosensors built using the same strategy, this work demonstrates the promise of an adaptable template for the detection of clinically relevant molecules in a variety of disease states. Ultimately, the biosensor presented here could improve upon current diagnostic methods for galactosemia providing fast and accurate measurements with clinical implications.

## Figures and Tables

**Table 1:** Typical Galactose Sensitivity Parameters—PU-Capped Xerogels Systems.

Xerogel System	*n*	Sensitivity ^a^ (μA/mM)	Response Time ^b^ (s)	Linear Range ^c^ (mM)	LOD ^d^ (mM)
OTMS	8	0.055 (±0.011)	44 (±6)	14 (±1)	0.06 (±0.01)
PTMS	11	0.049 (±0.011)	49 (±4)	16 (±2)	0.16 (±0.20)
IBTMS	5	0.105 (±0.020)	39 (±5)	13 (±1)	0.04 (±0.02)
PhTMS	4	0.056 (±0.013)	63 (±1)	18 (±1)	0.09 (±0.03)

Notes: Uncertainty represents standard error; ^a^ Sensitivity determined from linear regression of calibration. curves from 0 to 10 mM; ^b^ Response time calculated for time it takes to reach 95% of total response; ^c^ Linear range can vary sensor-to-sensor, typical range is reported; ^d^ LOD determined from 3·σ_blank_/sensitivity(slope).

## Supplementary Materials

The following are available online at http://www.mdpi.com/2079-4991/9/1/42/s1. Figure SM-1: *I-t* curves of OTMS system with galactose and catalase injections; Figures SM-2–4: *I-t* and calibration curves of galactose injections at OTMS, PTMS, IBTMS systems; Figure SM-5: % function of OTMS, PTMS, and IBTMS systems over time; Figures SM-6 and SM-7: galactose sensitivity of OTMS and PTMS as well as ODTMS, MTMS, and PhTMS as a function of pH, drying time, and GaOx concentration; Experimental details of UV-Vis and BSA assay measurements including spectrum of GaOx solution (Figure SM-8); Figure SM-9: *I-t* curves for OTMS and PTMS with and without PU capping layer during interferent injections; Figures SM-10 and SM-11: *I-t*, calibration curves and interferent responses with selectivity coefficients for IBTMS systems with polymer layers and CNT enhancement; Figure SM-12: *I-t* curve for interferents at films with gold nanoparticles at the solution interface and calibration and testing of galactose in spiked blood serum samples; Table SM-1: Table of biosensing performance parameters from literature.
