# Supplementary material for: First Generation Amperometric Biosensing of Galactose with Xerogel-Carbon Nanotube Layer-By-Layer Assemblies
Source: Nanomaterials (Basel). 2018 Dec 29;9(1):42. doi: 10.3390/nano9010042 (PMC6359589; doi:10.3390/nano9010042)
Supplement: Supplementary file 1 [file nanomaterials-09-00042-s001.pdf]

Supplementary Material

First Generation Amperometric Biosensing of Galactose with Xerogel-Carbon Nanotube Layer-by-Layer Assemblies

Najwa Labban,[1,†] Mulugeta B. Wayu,[1,†] Ciara M. Steele,[2] Tess S. Munoz,[1] Julie A. Pollock,[1] William S. Case,[2,*] and Michael C. Leopold[1,*]

[1] *Department of Chemistry, Gottwald Center for the Sciences, University of Richmond, Richmond, Virginia, 23173*
[2] *Department of Chemistry, Phifer Hall, Converse College, Spartanburg, South Carolina,* 29302

CONTENTS:

- **Figure SM-1.** Amperometric I-t curve during galactose and catalase injections at GaOx-doped OTMS xerogels.
- **Figure SM-2.** Representative I-t curves during galactose injections at GaOx-doped OTMS xerogels with and without PU capping layer.
- **Figure SM-3.** Representative I-t and calibration curves for galactose injections at GaOx-doped PTMS xerogels with and without PU capping layer.
- **Figure SM-4.** Representative I-t and calibration curves for galactose injections at GaOx-doped IBTMS xerogels with and without PU capping layer.
- **Figure SM-5.** Comparison of % function or stability with OTMS, PTMS, and IBTMS xerogel systems with and without PU.
- **Figure SM-6**. Galactose sensitivity comparisons as a function of pH, GaOx concentration, and xerogel drying times for OTMS and PTMS xerogel systems.
- **Figure SM-7.** Galactose sensitivity comparisons as a function of pH, GaOx concentration, and xerogel drying times for ODTMS, MTMS, and PhTMS xerogel systems.
- **Experimental details:** UV-Spectroscopy, BCA assay analysis of xerogel films with and without PU capping layer.
- **Figure SM-8.** UV-Vis spectra of GaOx (~14 mg/mL) solution (control experiment).
- **Figure SM-9.** Amperometric I-t curves of GaOx-doped xerogel films of **(A)** OTMS and **(B)** PTMS both with **(a)** and without **(b)** a PU capping layer during interferent injections.
- **Figure SM-10.** Amperometric I-t curve and corresponding calibration curve for IBTMS systems with CNT enhancement (without Nafion membrane).
- **Figure SM-11.** Amperometric I-t curve tracking interferent response and selectivity coefficients for IBTMS systems with CNT enhancement (without Nafion membrane).
- **Figure SM-12.** Amperometric I-t curve of Pt/DBR/COOH-SWCNT (1% Nafion)/IBTMS(GaOx)/PU/PU w/ CS-NPs during injections of common interferents.
- **Figure SM-13.** Amperometric I-t curve of Pt/DBR/COOH-SWCNT (1% Nafion)/IBTMS(GaOx)/PU w/ Nafion during calibration for galactose in serum and testing in galactose spiked blood serum samples.
- **Table SM-1.** Table of comparison for literature reports of galactose biosensing

* To whom correspondence should be addressed. Email: mleopold@Richmond.edu or will.case@converse.edu,

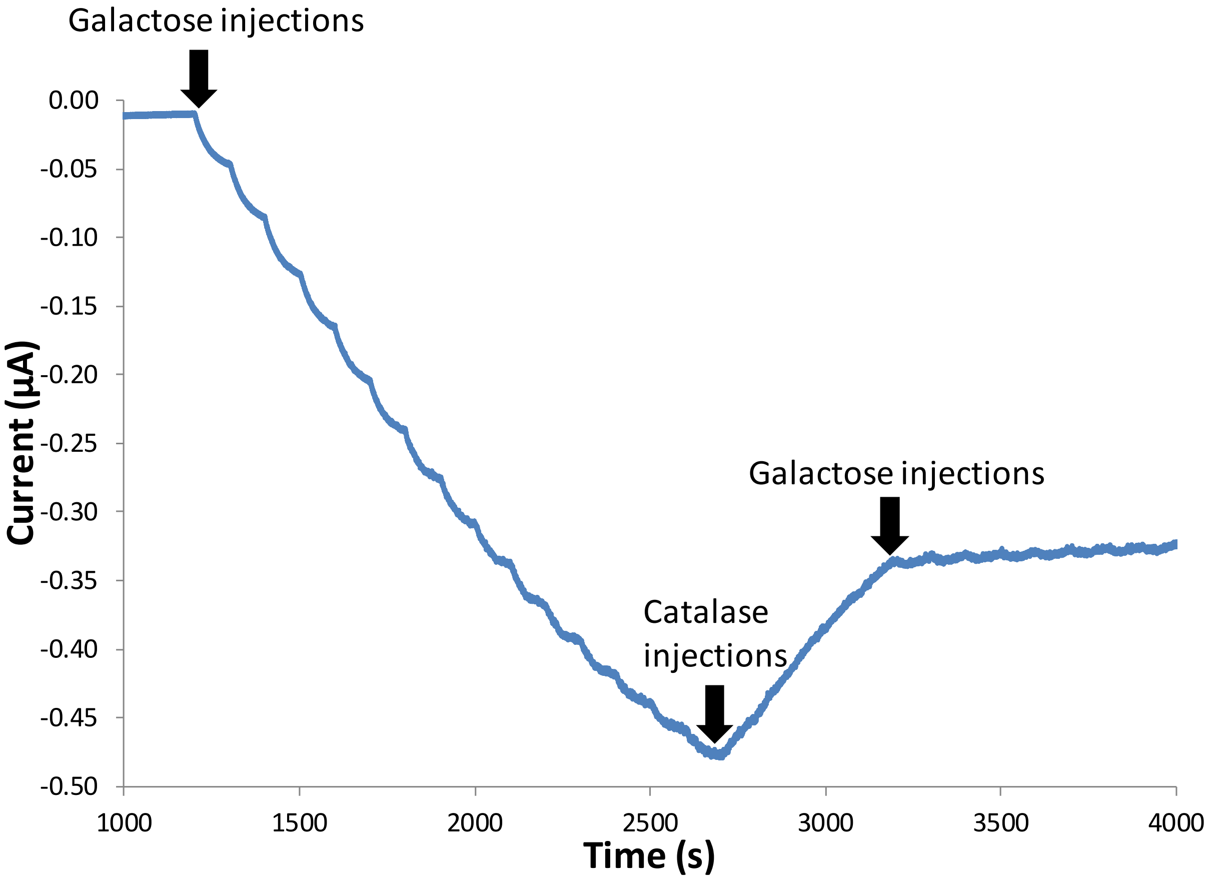

Figure SM-1. Amperometric I-t curve during successive 25 µL injections of galactose (1 mg/mL) or catalase (1 mg/mL) at a platinum electrode modified with a single layer of OTMS xerogel embedded with GaOx. Solution conditions: 8 mM potassium phosphate buffer (pH=7; stirred).

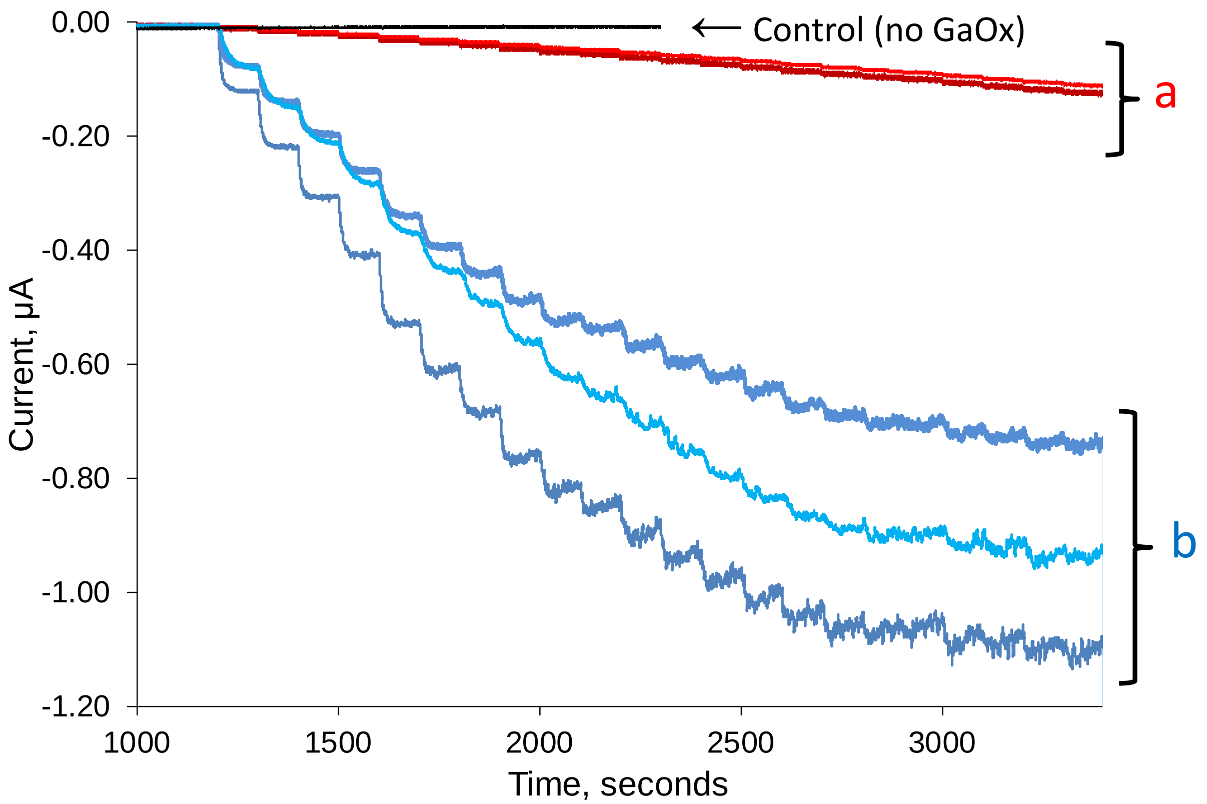

Figure SM-2. Representative **(A)** Amperometric I-t curves and **(B)** corresponding calibration curve analysis during 1 mM injections of galactose at **(a)** Pt/OTMS (GaOx) (n=3) and **(b)** Pt/OTMS GaOx)/PU (n=3). The I-t responses of these systems are also compared to a control system without enzyme and PU capping layer (Pt/OTMS - no GaOx). Solution conditions: 8 mM PBS (pH 7)

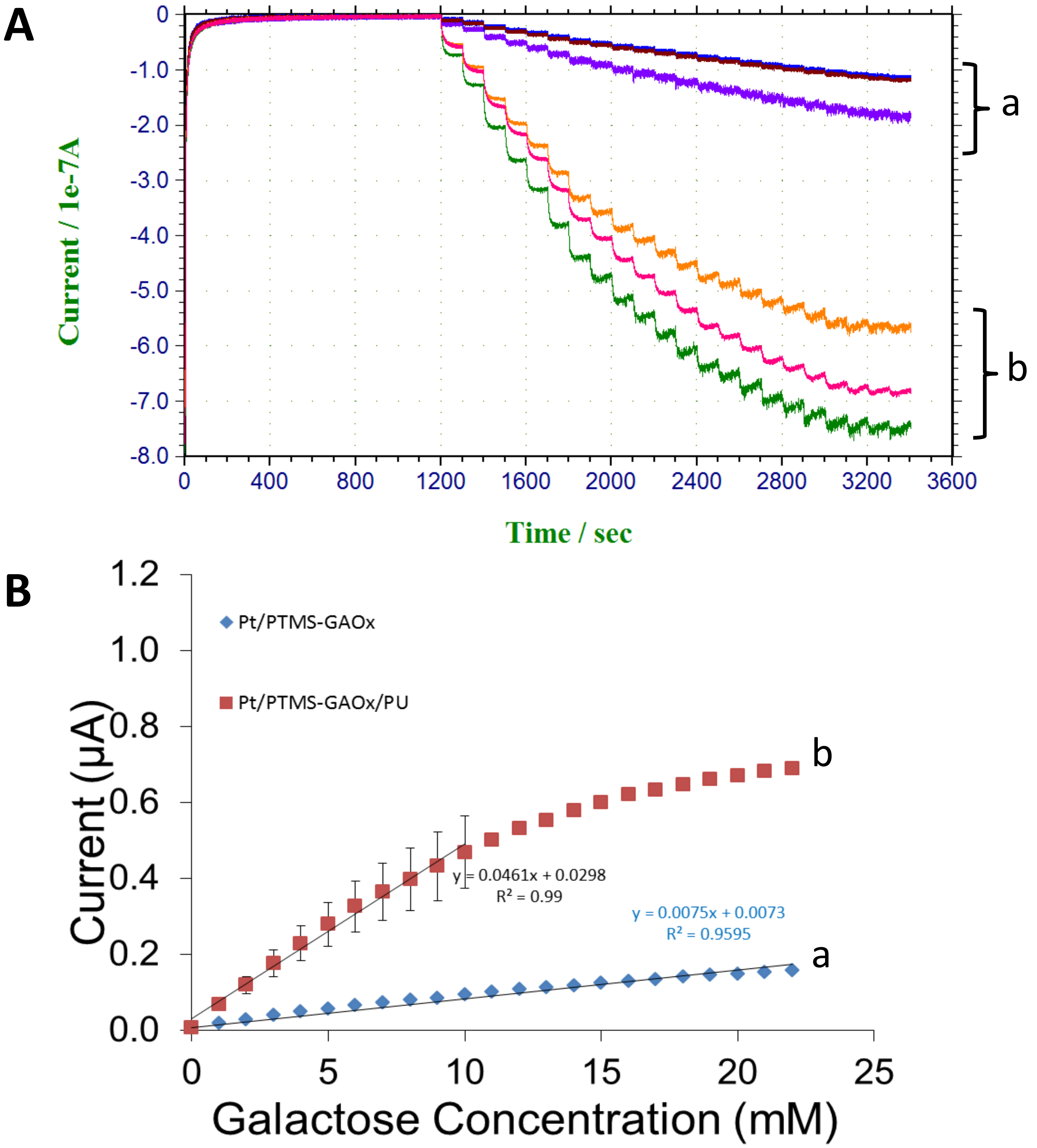

Figure SM-3. Representative **(A)** amperometric I-t curves and **(B)** corresponding calibration curve analysis during 1 mM injections of galactose at **(a)** Pt/PTMS (GaOx) (n=3); **(b)** Pt/PTMS GaOx)/PU (n=3). Solution conditions: 8 mM PBS (pH 7); Error bars represent standard deviation (n=3, each type of film). Note: In some cases, error bars are smaller than markers.

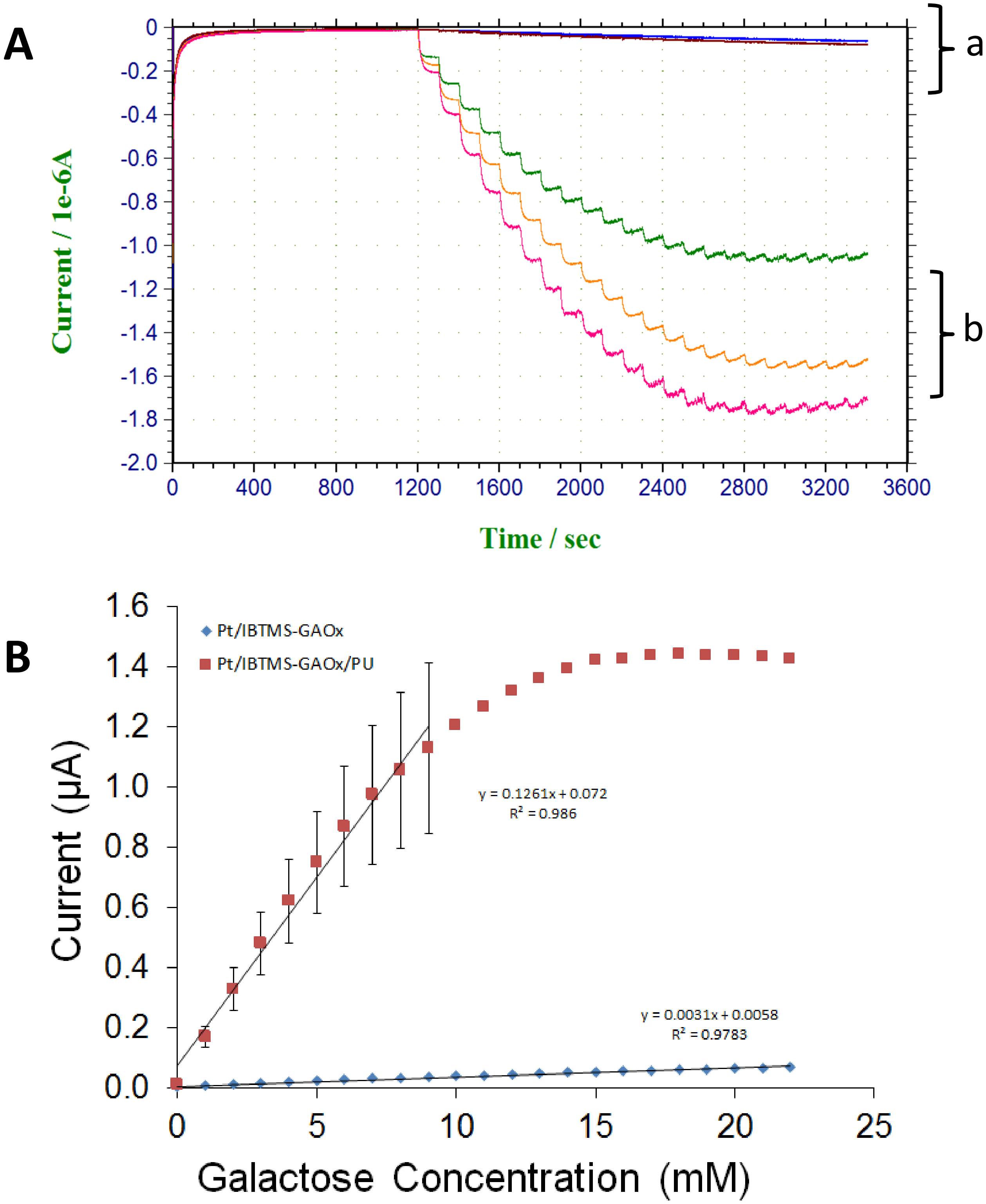

Figure SM-4. Representative **(A)** amperometric I-t curves and **(B)** corresponding calibration curve analysis during 1 mM injections of galactose at **(a)** Pt/IBTMS (GaOx); **(b)** Pt/IBTMS GaOx)/PU. Solution conditions: 8 mM PBS (pH 7); Error bars represent standard deviation (n=3, each type of film). Note: In some cases, error bars are smaller than markers.

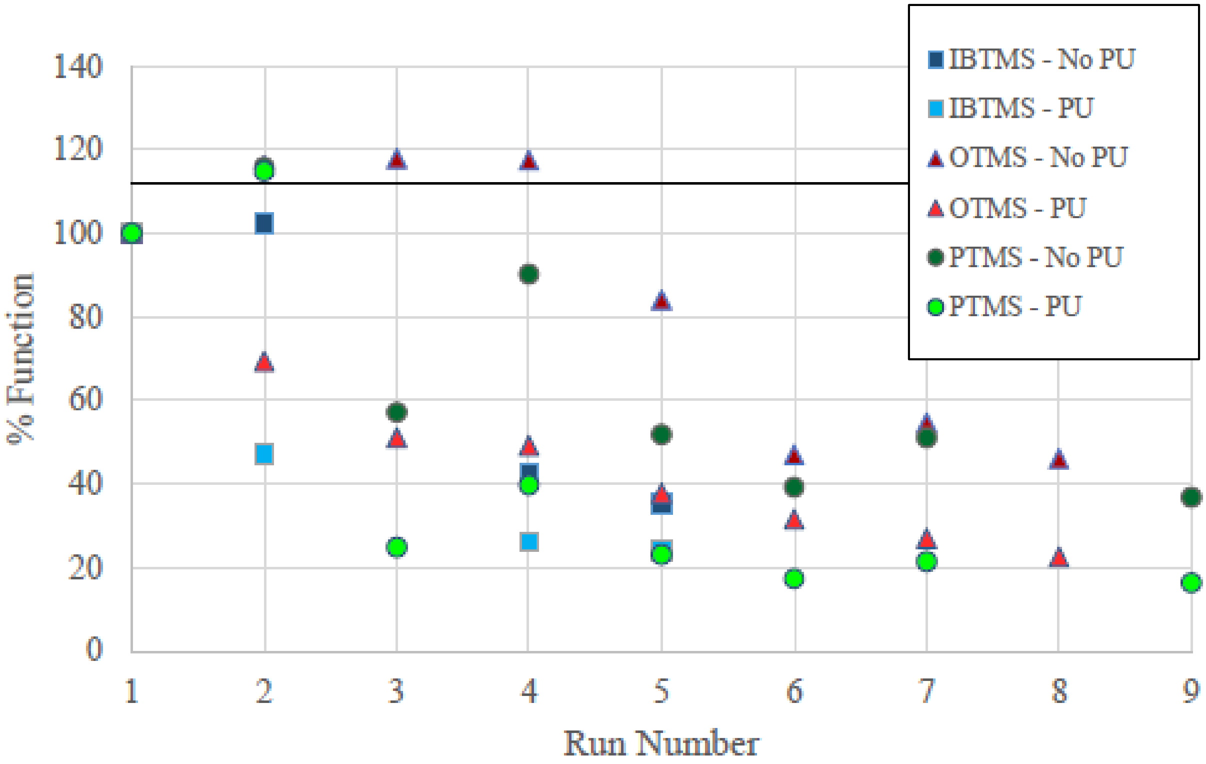

Figure SM-5. Comparison of % function or stability for electrodes modified with OTMS, PTMS, and IBTMS xerogels with and without the PU capping layer.

Figure SM-5

A

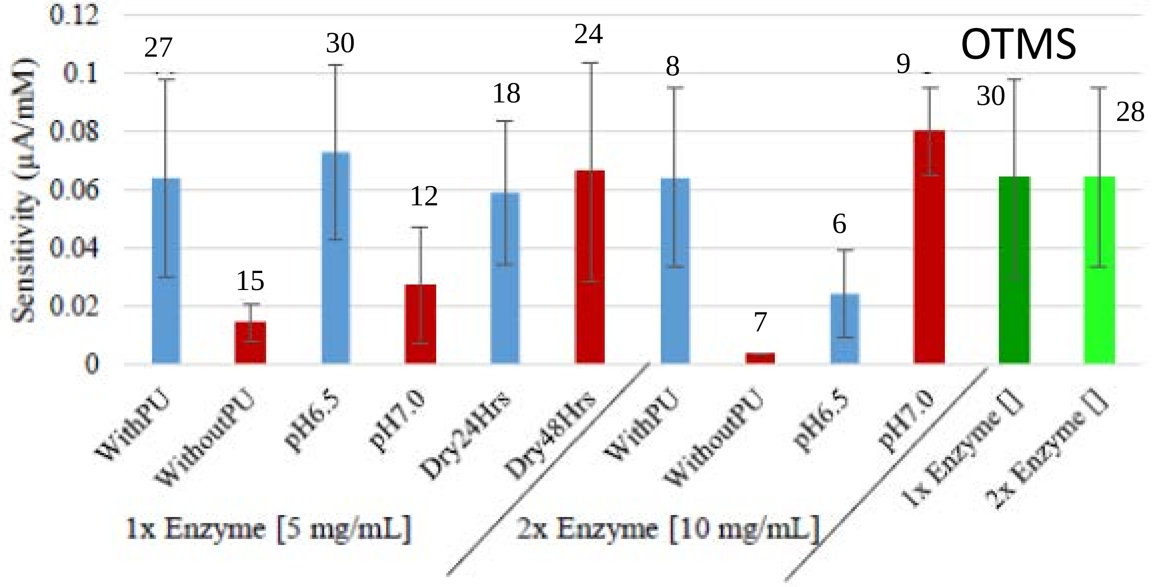

B

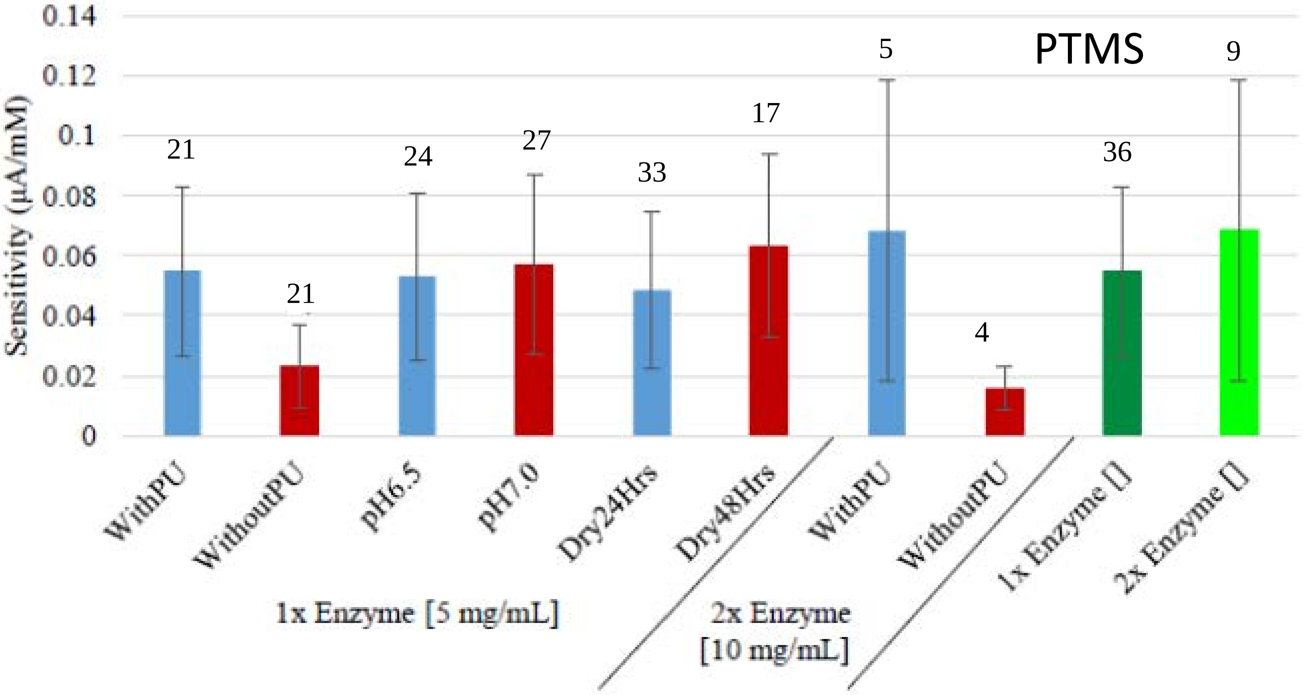

Figure SM-6. Comparison of pH, GaOx concentration, and drying time effects on sensitivity of electrodes modified with xerogel layers exhibiting PU signal enhancement effect, including **(A)** OTMS and **(B)** PTMS. **Note:** The enzyme concentrations reference here refer to the final concentrations within the 3 µL aliquots deposited onto the working electrode surface; values of n are shown above each result.

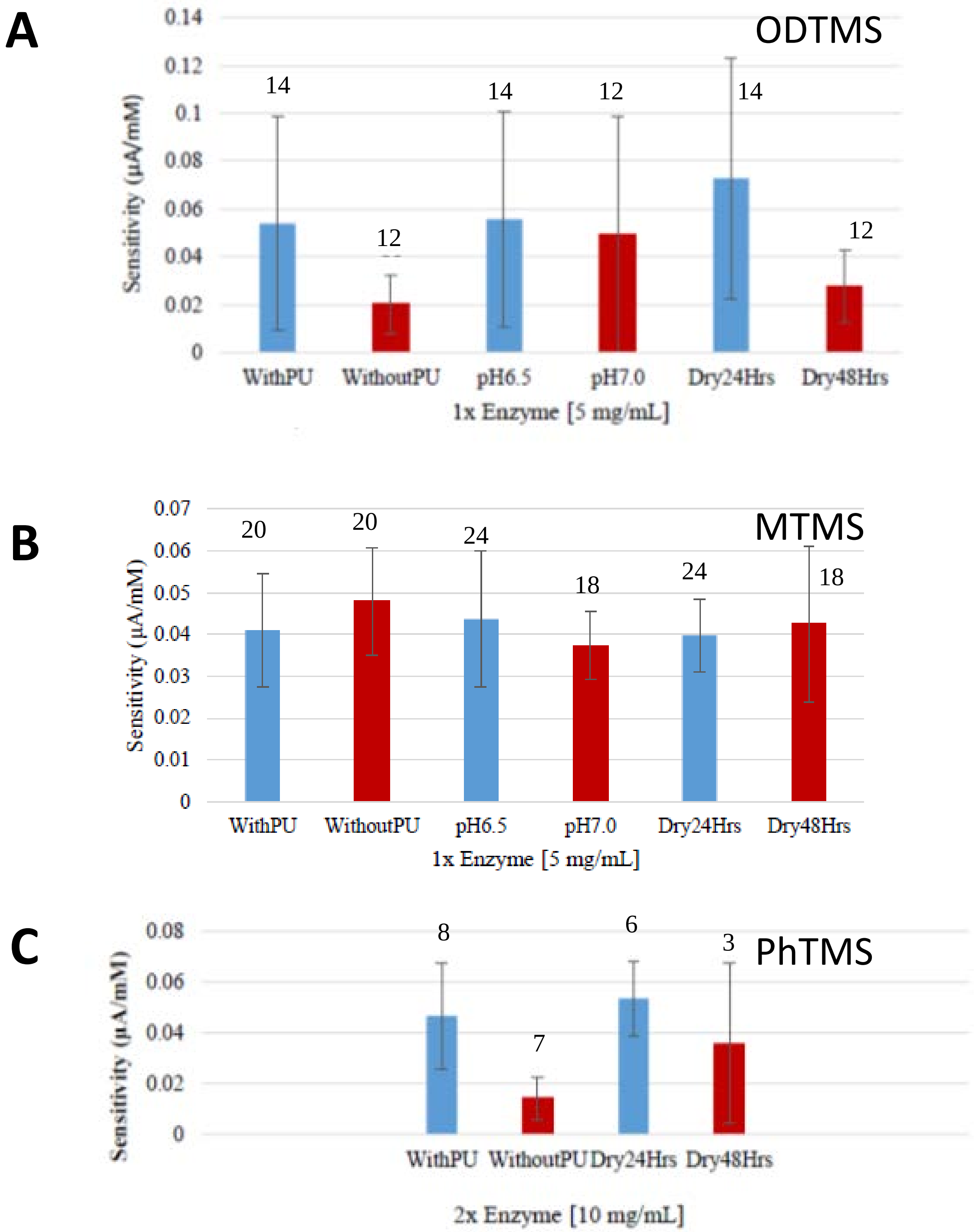

Figure SM-7. Comparison of pH, GaOx concentration, and drying time effects on sensitivity of electrodes modified with xerogel layers exhibiting PU signal enhancement effect, including **(A)** ODTMS, **(B)** MTMS, and **(C)** PhTMS. Note: The enzyme concentrations reference here refer to the final concentrations within the 3 µL aliquots deposited onto the working electrode surface; values of n are shown above each result..

Additional Experimental Details:

UV-Vis Spectroscopy. A 56.4 mg/mL solution of galactose oxidase (GaOx) was made by adding 0.014g of galactose oxidase to 250 µL in a centrifuge tube. A solution of 100 µL of tetrahydrofuran and 25 µL the specific silane. After vortexing the two tubes, 50 µL of the GaOx solution was added to the solution of tetrahydrofuran and silane. After additional vigorous vortexing of the mixture, a 3 µL were deposited on the glass slide. The slide was allowed to sit until the surface appeared dry and then another 3 µL film was deposited. This was repeated until there was five layers of the film on the glass slide in order to generate a film thick enough to be measureable with UV-Vis spectroscopy. The above process was done for both trimethoxymethylsilane (MTMS) and trimethoxy(octadecyl)silane (OTMS). For the blanks, five layers of either MTMS or OTMS were deposited on two separate glass slides. Initial measurements (0 minutes) were made on dry films before both blank (no enzyme silane films) and enzyme doped films were soaked in 8 mM phosphate (pH=6.5). The enzyme doped films were then measured every 5 minutes up to 30 minutes. Undoped xerogel films are nearly transparent and show no significant absorbance while solutions of GaOx exhibit broad spectrum absorbance (Supplementary Materials, Figure SM-8). The xerogel films are visible after deposition, drying/aging and remain visible after soaking (Figure 4).

BCA Assay

Sample Preparation

1) <u>MPTMS +GaOx</u>: 50uL of a vortexed GaOx solution (9.4mg in 250uL of water) was added to a vortexed mixture of 25uL of MPTMS and 100uL of tetrahydrofuran. The resulting solution was vortexed and 3uL were dropcast onto 6
electrodes that had been polished and electrochemically cleaned. After 24 hours of drying at 50% humidity, 10uL of polyurethane (50:50 blend of HPU and TPU) were added to 3 of the electrodes and allowed to dry for 30 minutes in ambient air.

2) <u>MTMS +GaOx</u>: Procedure 1 was repeated using MTMS.
3) <u>OTMS +GaOx</u>: Procedure 1 was repeated using OTMS.
4) <u>IBTMS +GaOx</u>: Procedure 1 was repeated using IBTMS.

All electrodes were then soaked in microcentrifuge tubes containing 250uL of potassium phosphate buffer (8mM, pH 7) for one hour. 25uL of the solution in each tube were added to a well in a clear assay plate. A standard curve of GaOx was generated by the serial 2X dilution of a 2mg/uL GaOx six times (for a total of seven concentrations). 200uL of BCA working reagent were added to each well and the plate was incubated at 37C for 30 minutes before being read at 562 nm.

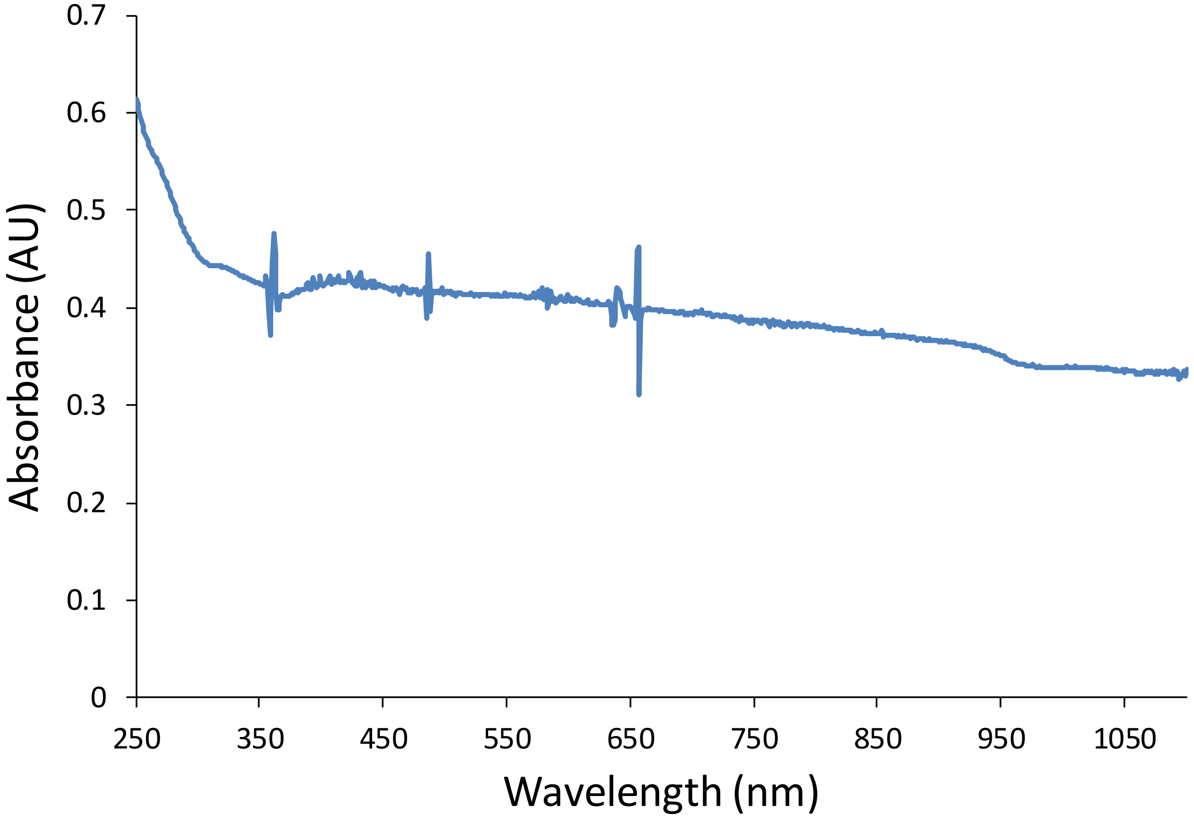

Figure SM-8. UV-Vis spectra of GaOx (~14 mg/mL) solution (control experiment).
Note: Xerogel films are visible on the electrode both before and after soaking (Figure 4).

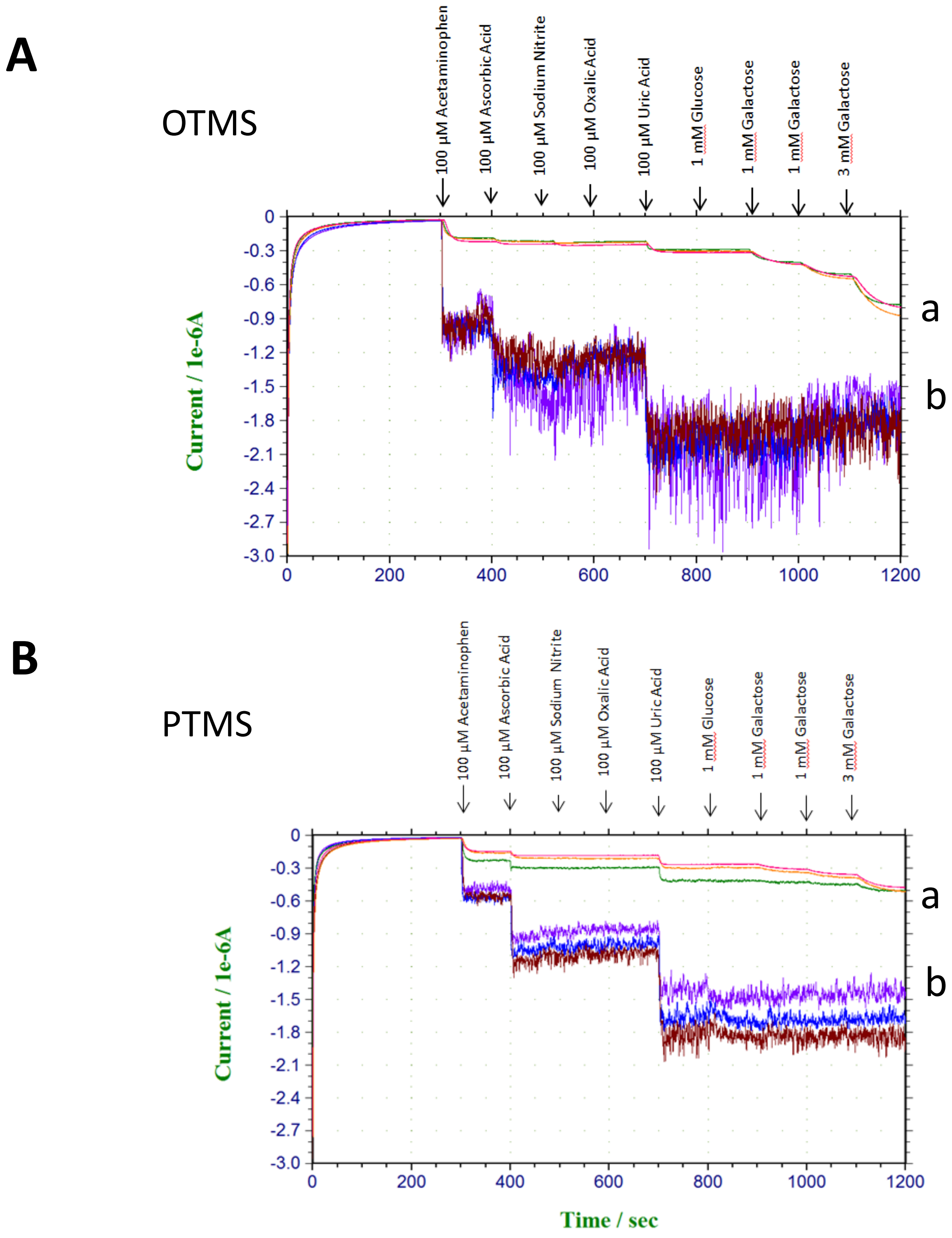

Figure SM-9. Amperometric I-t curves of GaOx-doped xerogel films of **(A)** OTMS and **(B)** PTMS both **(a)** with and **(b)** without a PU capping layer during common interferent injections. Note: n=3 in each case; acetaminophen is commonly tested as an interferent but is a synthetic species vs. the other endogenous species.

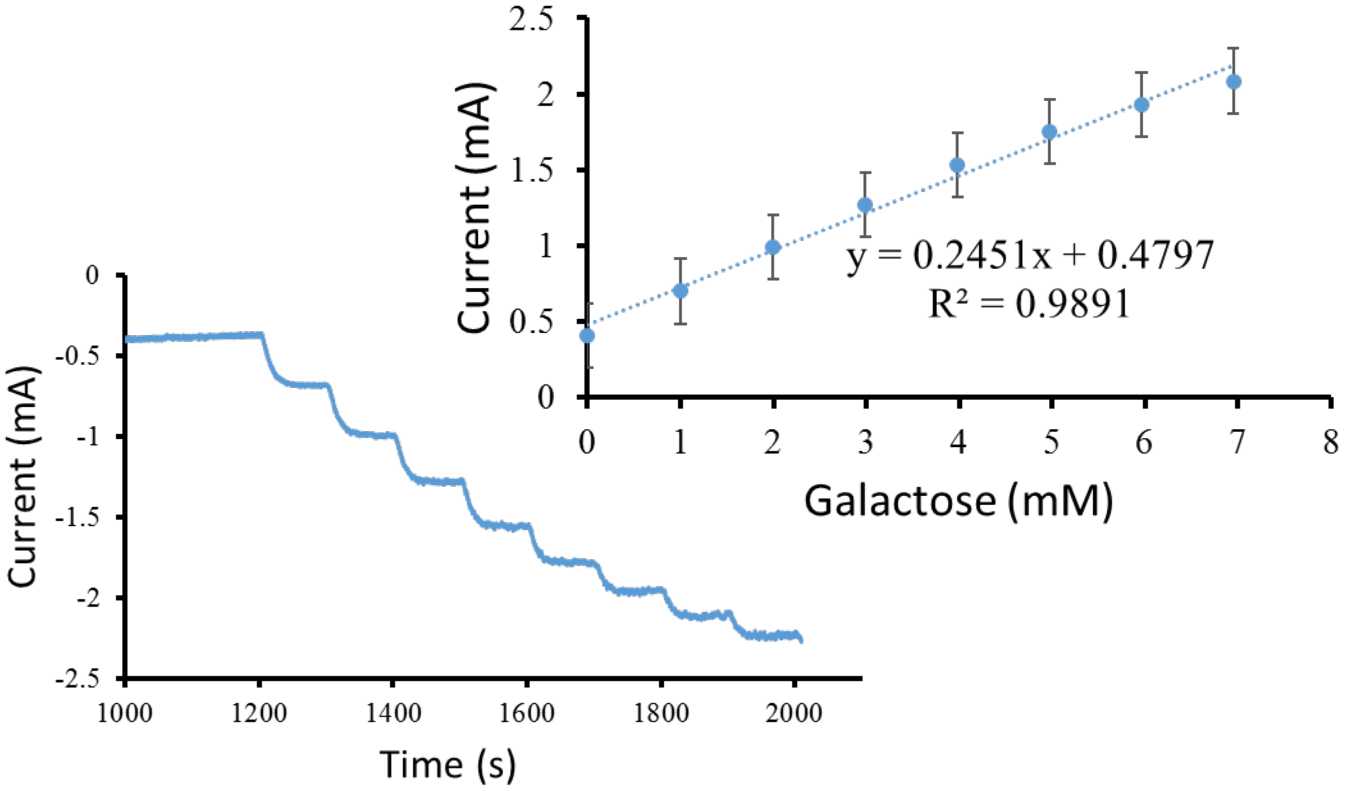

Figure SM-10. Representative amperometric I-t curves and corresponding calibration curves (insets) of Pt/DBR/COOH-SWCNT/IBTMS(GaOx)/PU (no Nafion) during 1 mM injections of galactose. Note: In some cases, error bars are smaller than markers (n=4).

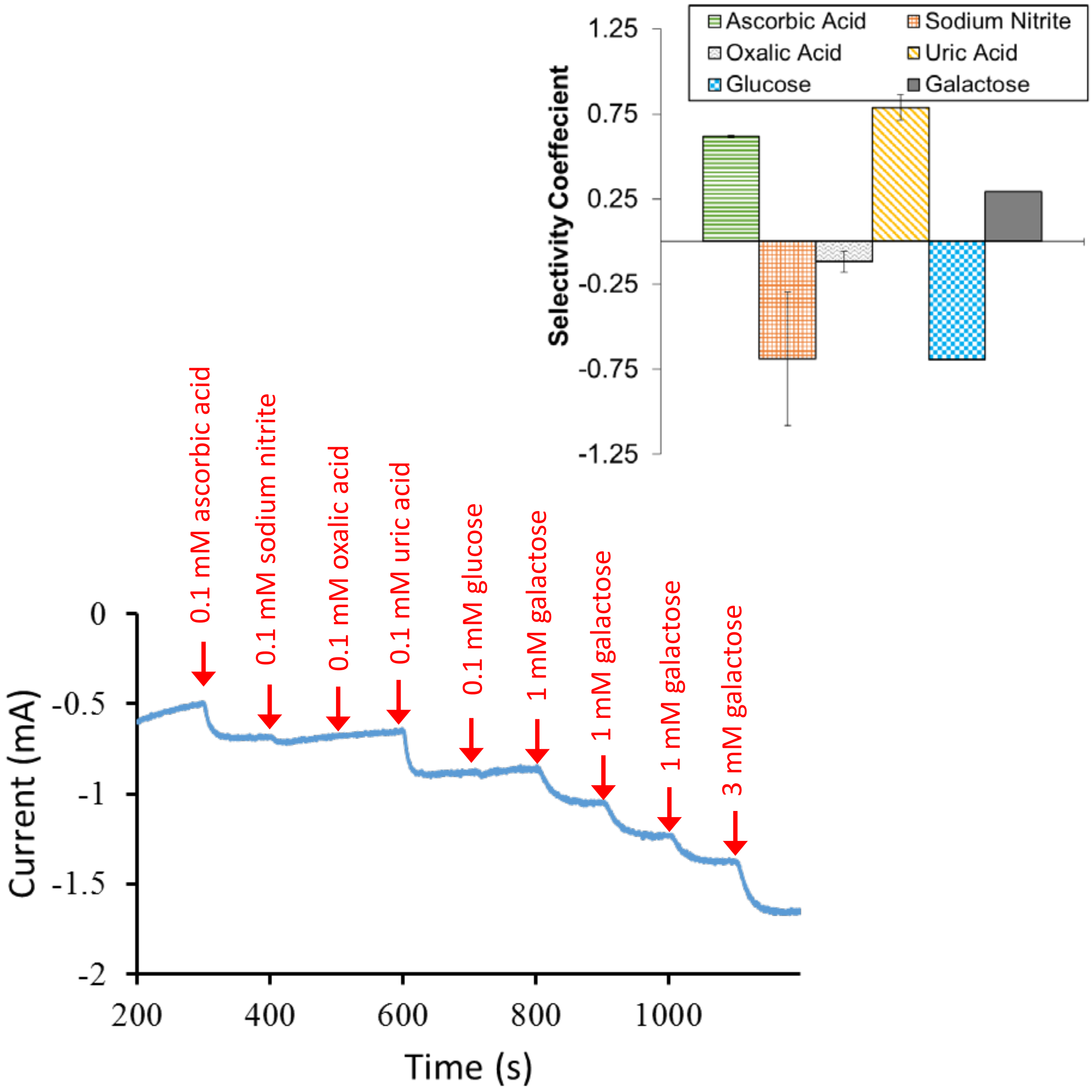

Figure SM-11. Representative amperometric I-t curves and corresponding selectivity coefficient graphs (**insets**) of Pt/DBR/COOH-SWCNT/IBTMS(GaOx)/PU (no Nafion) during injections of common interferent species and galactose in 100 second intervals.

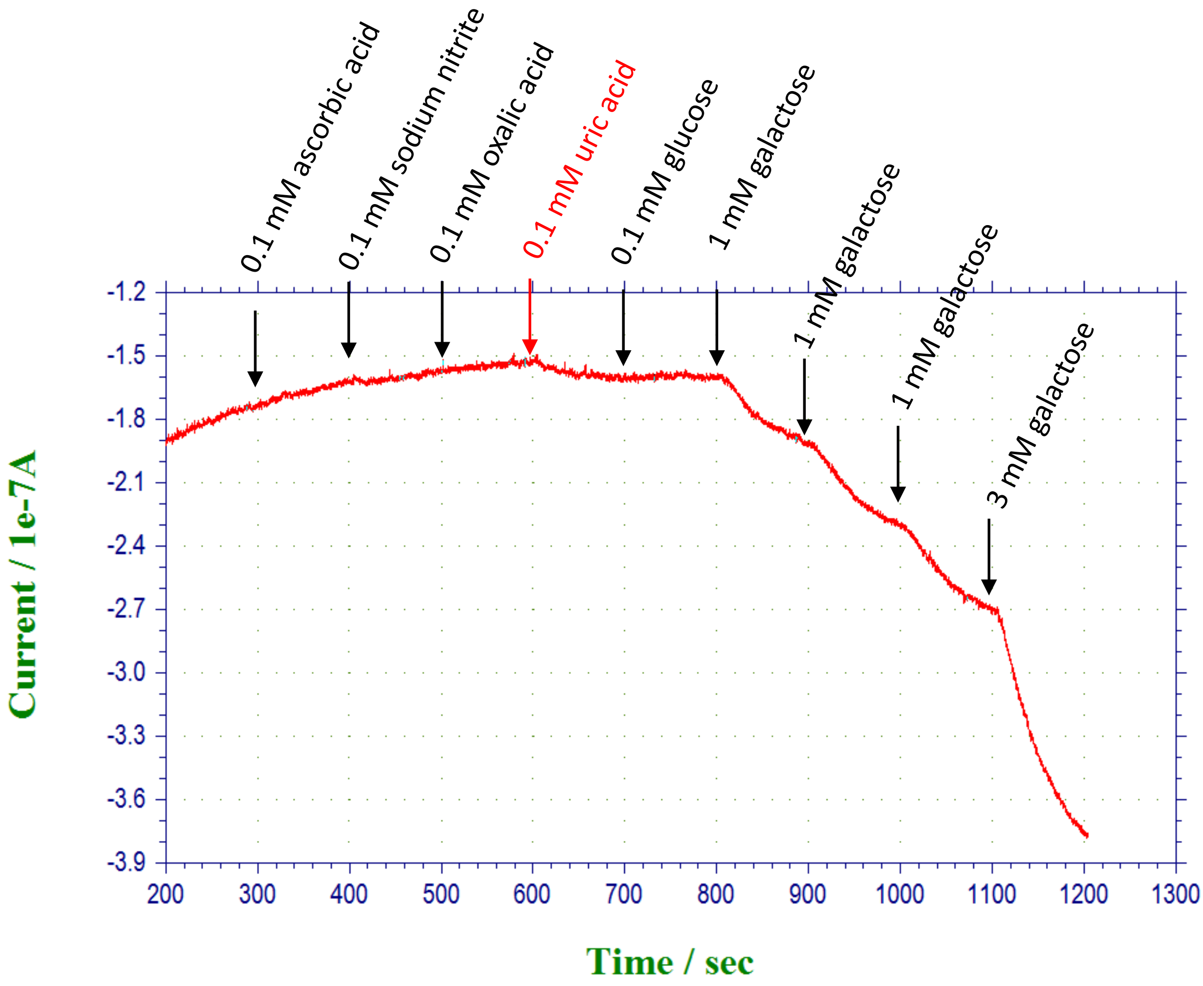

Figure SM-12. Representative amperometric I-t curve of Pt/DBR/COOH-SWCNT (1% Nafion)/IBTMS(GaOx)/PU/PU w/ CS-NPs during injections of common interferent species and galactose in 100 second intervals.

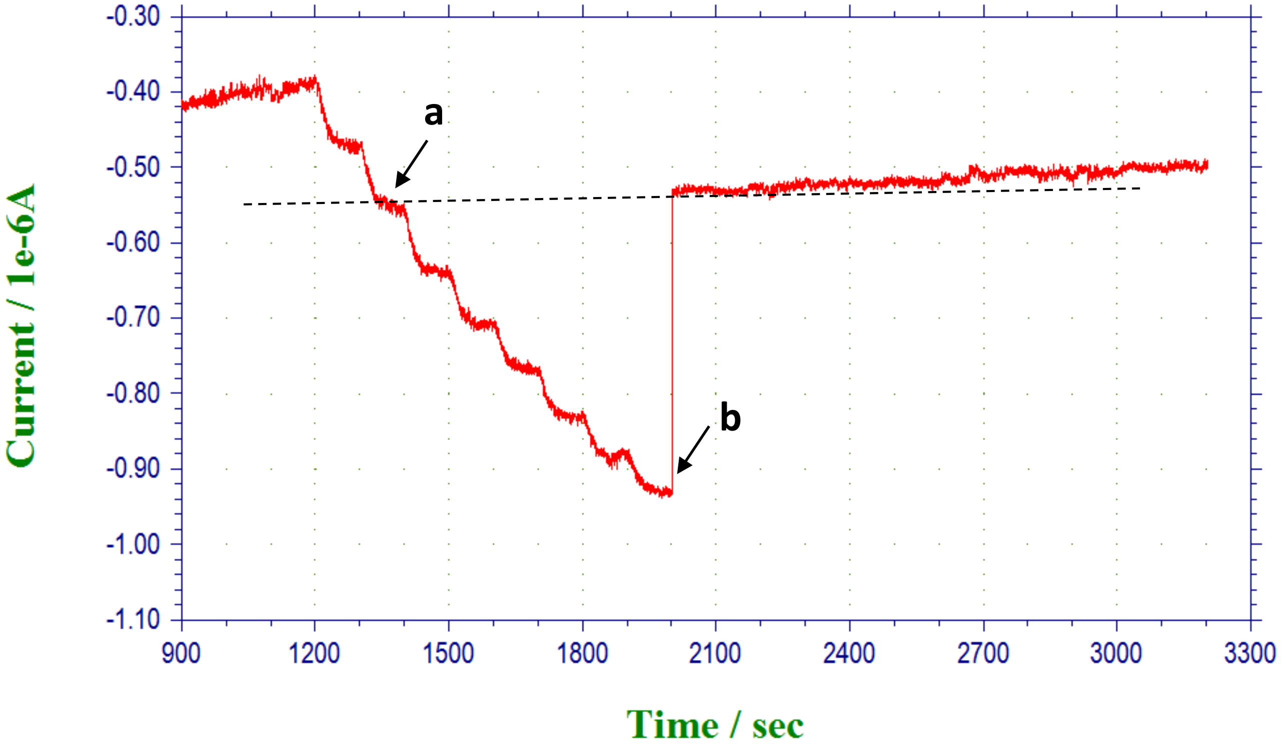

Figure SM-13. Representative amperometric I-t curve of Pt/DBR/COOH-SWCNT (1% Nafion)/IBTMS(GaOx)/PU w/ Nafion during (a) standard injections of 1 mM galactose in serum samples for calibration and (b) after pausing amperometry, rinsing the electrodes, and then re-immersing them in a serum sample spiked with 2 mM galactose.

Table SM-1. Comparison of Galactose Biosensor Performance Parameters – Literature Comparison

System	Type	WE	Sensitivity ($\mu A/mM.cm^2$)	Response Time (s)†	Linear Range (mM)	LOD‡ (μM)	Stability	Ref
PU/GaOx-IBTMS/COOH-SWCNT/ Poly(1,3-DAB/Res /Pt	1st G (H_2O_2)	Pt	0.037 (±0.006)	30 (±1.3)	0 to 7	78	--	Current Work
RGO-Au@PLLA/GAox/GCE	1st G (H_2O_2)	GCE	N/A	N/A	0.0015 to 0.522	0.79	4% decrease after 10 days	[23]
Carbon/Pt/Poly(1,3-DAB/Res/Nafion/GAO	1st G (H_2O_2)	Carbon/ Pt	N/A	18	0.05 to 6.0	50	>30 days	[24]
Pt/p(HEMA)PPy/GalOx	1st G (H_2O_2)	Pt	N/A	70	0.05 to 10.00	25	30% decrease after 9 months	[25]
P3HT/SA/GaO/ITO	1st G (H_2O_2)	ITO	N/A	60	55.51 to 222.02	55510	Upto 45°C, same response upto 20 days	[26]
Pt/PEG-GAO-PPy	1st G (H_2O_2)	Pt	0.106	< 40	0 – 24	N/A	Upto 35°C	[27]
GCE/CHIT-SWCNT/GalOD/NAF(P)	1st G (H_2O_2)	GCE	1.126	N/A	0.025 to 1.00	25	Response decreased only by 2% after continuous use for 2.5h	[28]
Pt/Poly(GMA-co-VFc)/GAox	2nd G	Pt	0.023	5	2000 to 20000	100.00	Stable upto 35°C; Response decreased only by 7% after 8 days; 40% after 30 days	[29]
GCE/Co3O4-MWCNTs-CS/GaOx/Nafion	1st G (H_2O_2)	GCE	0.79	20	9.0×10^{-3} to 1.00	0.90	N/A	[30]
Au/PEP/GaOx	1st G (H_2O_2)	Au	1.75	5	2.0 to 16	25	Same response upto 10 days	[31]

Additional notes: Polyurethane (PU), Isobutyl(trimethoxy)silane (IBTMS), Diaminobenzene/Resorcinol (DAB/Res), reduced graphene oxide (RGO), poly L-Lactide (PLLA), poly(2-hydroxyethyl methacrylate) (pHEMA), poly-pyrole (PPy), poly(3-hexyl thiophene)/stearic acid (P3HT/SA), indium tin–oxide (ITO), poly(ethylene glycol) (PEG), glycidyl methacrylate-co-vinylferrocene (GMA-co-VFc), Chitosan (CS), Poly(N-glycidylpyrrole-co-pyrrole (PEP); †Calculated from 95% of total response; ‡Calculated from $3\sigma_{blnk}$ /slope of calibration curve.
